# The Hos2 Histone Deacetylase Controls *Ustilago maydis* Virulence through Direct Regulation of Mating-Type Genes

**DOI:** 10.1371/journal.ppat.1005134

**Published:** 2015-08-28

**Authors:** Alberto Elías-Villalobos, Alfonso Fernández-Álvarez, Ismael Moreno-Sánchez, Dominique Helmlinger, José I. Ibeas

**Affiliations:** 1 Centro Andaluz de Biología del Desarrollo, Universidad Pablo de Olavide, de Sevilla-Consejo Superior de Investigaciones Científicas-Junta de Andalucía, Sevilla, Spain; 2 Centre de Recherche de Biochimie Macromoléculaire, Centre National de la Recherche Scientifique UMR5237-Université de Montpellier, Montpellier, France; Karlsruhe Institute of Technology, GERMANY

## Abstract

Morphological changes are critical for host colonisation in plant pathogenic fungi. These changes occur at specific stages of their pathogenic cycle in response to environmental signals and are mediated by transcription factors, which act as master regulators. Histone deacetylases (HDACs) play crucial roles in regulating gene expression, for example by locally modulating the accessibility of chromatin to transcriptional regulators. It has been reported that HDACs play important roles in the virulence of plant fungi. However, the specific environment-sensing pathways that control fungal virulence via HDACs remain poorly characterised. Here we address this question using the maize pathogen *Ustilago maydis*. We find that the HDAC Hos2 is required for the dimorphic switch and pathogenic development in *U*. *maydis*. The deletion of *hos2* abolishes the cAMP-dependent expression of mating type genes. Moreover, ChIP experiments detect Hos2 binding to the gene bodies of mating-type genes, which increases in proportion to their expression level following cAMP addition. These observations suggest that Hos2 acts as a downstream component of the cAMP-PKA pathway to control the expression of mating-type genes. Interestingly, we found that Clr3, another HDAC present in *U*. *maydis*, also contributes to the cAMP-dependent regulation of mating-type gene expression, demonstrating that Hos2 is not the only HDAC involved in this control system. Overall, our results provide new insights into the role of HDACs in fungal phytopathogenesis.

## Author Summary

Many pathogenic fungi need to undergo morphological changes in order to infect their hosts. Typically, pathogenic fungi switch from a non-pathogenic yeast-like form to a polarised pathogenic filament. This morphological switch is regulated genetically and is triggered by specific environmental conditions. Histone deacetylases (HDACs) are important regulators of chromatin structure and gene expression. In this study, we investigate the role of HDACs as targets of the signalling pathways that activate fungal virulence programs in response to specific external signals. We identify two specific HDACs, Hos2 and Clr3, that are required for the virulence of the corn smut fungus, *Ustilago maydis*. Our results reveal that Hos2 and Clr3 function in the cAMP-PKA cascade, a nutrient-sensing pathway conserved between all eukaryotes.

## Introduction

The switch between yeast and hypha stages, or dimorphism, is a key morphological conversion required for the virulence of several animal and plant pathogenic fungi [1–4]. This process occurs at specific stages of fungal infection and is tightly controlled. The two best studied signalling pathways regulating gene expression during dimorphism are the mitogen activated protein (MAP) kinase cascade and the cyclic-AMP protein kinase A (cAMP-PKA) pathway. Their activation is typically controlled by specific environmental stimuli and results in the induction of master regulatory genes [5–8]. *Ustilago maydis* is a well-established model organism for the study of the yeast-hypha dimorphic switch, a key stage of its pathogenic cycle [6, 8, 9]. The *U*. *maydis* pathogenic cycle starts when two mating compatible haploid yeast cells recognise each other via a pheromone-receptor system. Mating leads to the formation of a dikaryon filament, whose apical tip differentiates into a specialised structure for plant penetration known as the appressorium [10–12]. Once inside the plant, *U*. *maydis* proliferates, inducing the formation of tumours and eventually develops into diploid spores [13]. Thus, the transition from the yeast form to the infective filamentous one is crucial for *U*. *maydis* pathogenicity.

The control of this process relies on a tetrapolar system consisting of the biallelic *a* and multiallelic *b* loci. The *a* locus encodes components of the pheromone-receptor system, allowing cells from opposite mating types to recognise each other, form conjugation tubes and fuse [14, 15]. The fate of the resulting dikaryon is then controlled by the *b* locus, which encodes two transcription factors, *bE* and *bW*. When different mating type-specific alleles of *bE* and *bW* are expressed, they form a compatible heterodimer that activates the filamentation and virulence programs [16, 17]. MAP kinase and cAMP-PKA pathways are necessary for sensing pheromone and environmental signals. Both pathways lead to the transcriptional and post-translational activation of the transcription factor Prf1 (Pheromone-responsive factor 1). Once activated, Prf1 binds to the promoters of mating-type gene loci and activates their expression (S1 Fig) [18–23]. Prf1 is a central regulatory factor during mating and virulence, and is tightly controlled at the transcriptional level [19, 20, 22, 24–27]. Chromatin structure and modifications are central to gene regulation, however, little is known about their contribution to the control of *prf1* and of Prf1-dependent gene expression.

Histone deacetylases (HDACs) are important regulators of gene expression and are grouped into different classes. Class I (*Saccharomyces cerevisiae* RPD3-like) and class II (HDA1-like) HDACs are conserved from yeast to humans. Typically, nuclear histone deacetylases inhibit transcription through the deacetylation of promoter-bound histones. However, several groups have reported HDACs with both activating and repressing functions [28–31]. For example, the *S*. *cerevisiae* Hos2/Set3 HDAC complex represses specific meiotic genes while activating others [28, 29].

HDACs have been implicated in many physiological processes, with their roles varying between different biological contexts. This is exemplified by the HDACs of plant and animal pathogenic fungi. There are important differences in the strategy employed by a fungus to colonise an animal or plant, and this seems to be reflected in the virulence-related process controlled by HDACs in each case. For example, Hos2 regulates the dimorphic switch of *Candida albicans* during animal infections [32], whereas it controls plant tissue penetration and post-penetration stages of plant pathogens [33, 34].

Finally, very little is known about the downstream HDAC gene targets required for plant pathogen virulence, or how HDAC activity integrates with the upstream signalling pathways that control pathogenic development. Here, we report a comprehensive characterisation of class I and II HDAC homologues in the plant pathogenic fungus *U*. *maydis*. Our results demonstrate that Hos2 is important for the yeast-to-hypha transition and fungal virulence. Analysis of deletion mutants and ChIP experiments strongly suggest that Hos2 directly regulates the expression of *U*. *maydis* mating-type genes downstream of the cAMP-PKA pathway. Lastly, we present evidence indicating that another HDAC, Clr3, functionally interacts with Hos2 in the regulation of mating-type genes through the cAMP-PKA pathway.

## Results

### Hos2 is important for *U*. *maydis* pathogenicity

**Fig 1:**
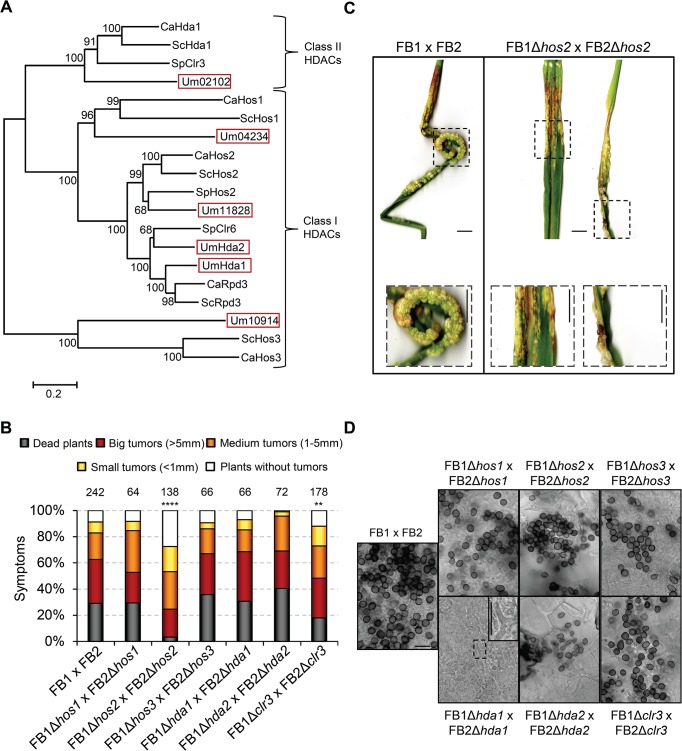
Hos2 is important for pathogenicity. (A) Phylogenetic tree of class I and II HDACs in *Saccharomyces cerevisiae* (Sc), *Schizosaccharomyces pombe* (Sp), *Candida albicans* (Ca) and *Ustilago maydis* (Um). The scale bar represents an evolutionary distance of 0.2 amino acid substitutions per site. (B) Quantification of plant symptoms infected with the indicated strains 14 days post-infection (dpi). Mean values of at least three independent experiments are shown. The total number of infected plants is indicated above each column. Statistically significant differences of each mutant cross compared to the wild-type cross are indicated (**** and ** denote a *p*<0.0001 and *p*<0.01 respectively, Mann-Whitney test) (C) Representative images of plants with infected wild-type or Δ*hos2* mutant strains 14 dpi. Scale bar = 1 cm. (D) Spore production, visualised by light microscopy, in the indicated HDAC mutants 21 dpi. Scale bar = 20 μm.

A BLAST search and phylogenetic analysis revealed that *Ustilago maydis* genome harbours six putative class I and II HDACs, a putative orthologue of each HDAC found in *Saccharomyces cerevisiae*, *Candida albicans*, and *Schizosaccharomyces pombe*, except for the Rpd3/Clr6 HDAC, for which we found two distinct homologues in *U*. *maydis* (Fig 1A). Two of the six *U*. *maydis* HDACs were already named: Hda1 for Um02065 [35] and Hda2 for Um11308 [36]. The other four proteins were named according to their closest relative in *S*. *cerevisiae* or *S*. *pombe*. Thus, we named Um04234 Hos1, Um11828 Hos2 (annotated in the MIPS *Ustilago maydis* database, MUMDB), Um10914 Hos3 and Um02102 Clr3. All four proteins possess the domains characteristic of their type as shown in S2 Fig.

To test the requirement of each HDAC for *U*. *maydis* virulence, we generated deletion mutants for class I and II HDACs in FB1 and FB2 mating-compatible strains. We then infected seven day-old maize seedlings with all the HDAC mutant and wild-type FB1 and FB2 strains. Symptoms were scored 14 days post infection (dpi). As shown in Fig 1B, most HDAC mutants showed wild-type pathogenic infection rates. However, Δ*hos2* mutants showed a strong reduction in pathogenesis. The absence of Hos2 results in an approximate 3-fold increase in the number of tumour-free plants, a 6-fold decrease in plant death and a decrease in the size of the developed tumours (Fig 1B and 1C), indicating that Hos2 is required for full *U*. *maydis* virulence. During this analysis, we also noticed a modest decrease in virulence when *clr3* was deleted, especially regarding the number of dead plants (Fig 1B).

Since *U*. *maydis* Hda1 is essential for teliospore development [35], we investigated whether other HDAC mutants were required for spore formation in this fungus. To this end, we analysed spore production inside maize tumours 21 days post infection. This analysis revealed that, besides Hda1, no other HDAC is required for spore formation (Fig 1D). To conclude, Hos2 and Hda1 control important yet distinct stages of the pathogenic cycle in *U*. *maydis*.

### *U*. *maydis* Hos2 is a *bona fide* HDAC

To confirm our sequence alignment analysis (Fig 1A), we sought to experimentally confirm that *U*. *maydis* Hos2 is indeed an HDAC. With this aim, we compared H4K16 acetylation, the canonical histone target of Hos2 [29, 37], in *U*. *maydis* Δ*hos2* mutant relative to the wild-type control. As can be observed in S3A Fig, the absence of Hos2 increased H4K16 acetylation levels confirming its HDAC activity. Additionally, we observed that a Δ*hos2* mutant shows a hypersensitivity response to Trichostatin A (TSA), as previously described in *S*. *pombe* [38, 39], (S3B Fig). These results confirm that we have identified the functional *U*. *maydis* Hos2 HDAC.

### Single HDAC deletion mutants show normal saprophytic growth but Hos2 is required for mating

**Fig 2:**
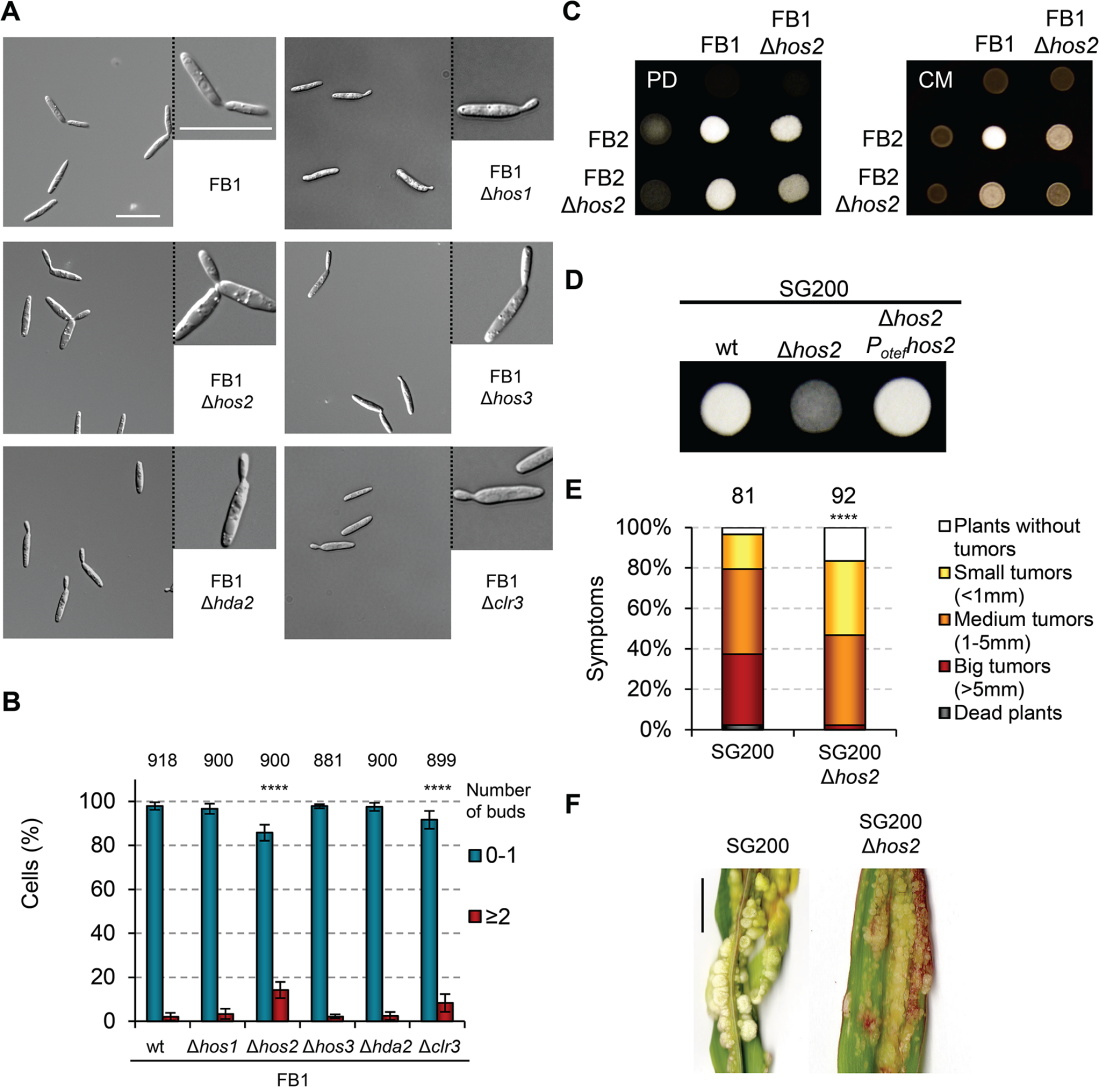
Hos2 is required for mating. (A) Light microscopy images of the indicated HDAC *Ustilago maydis* mutants in a FB1 background. Cells were grown in rich YEPSL media until exponential phase. (B) Quantification of the number of buds per cell in the indicated strains. Total number of cells counted for each strain is indicated above each pair of columns. Mean values and standard deviations (SDs) from three independent experiments are shown. Asterisks indicate statistically significant differences compared to wild-type (Fisher’s exact test. **** denotes a *p*<0.0001). (C) Mating crosses between wild-type FB1 and FB2 or Δ*hos2* mutant strains grown on charcoal-containing PD (left, PD) or CM (right, CM) plates for 24 hours at 25°C. (D) Filamentation of the indicated solopathogenic strains grown on PD charcoal plates for 24 h at 25°C. (E) Quantification of symptoms in maize plants infected with the indicated strains at 14 dpi. Mean values of three independent experiments are shown. The total number of infected plants is indicated above each column. **** indicates statistically significant difference with *p*<0.0001 (Mann-Whitney test). (F) Representative images of plants infected with SG200 or SG200Δ*hos2* as indicated. Scale bar = 1 cm.

To identify the cause of the reduced virulence shown by Δ*hos2* cells, we investigated the role of Hos2 at different stages of the *U*. *maydis* life cycle, comparing its behaviour to the other HDAC mutants. Δ*hda1* cells have previously been shown to have no phenotype except during spore formation, and were therefore not analysed further [35]. Light microscopy analysis of cells grown in liquid culture revealed no major anomalies, although we noticed that in an FB1 background 14% of Δ*hos2* and 8.3% of Δ*clr3* mutant cells were multi-budded, without significantly affecting their doubling time or cell shape during exponential growth (Figs 2A, 2B and S4A–S4C). Similar results were observed in an FB2 background (S4A, S4D and S4E Fig).

Mating between two compatible haploid partners is the first step of the *U*. *maydis* pathogenic cycle. To check whether Δ*hos2* mutants are able to mate normally, we used charcoal-containing plates, which mimic the plant leaf surface and induce the mating process. Mating between compatible partners and post-fusion filamentation can be visualised by the appearance of white and fuzzy colonies on charcoal plates. As shown in Fig 2C, mating between FB1Δ*hos2* and FB2Δ*hos2* mutants resulted in reduced white colony fuzziness relative to a cross of wild-type cells. Comparing rich (PD) and complete (CM) charcoal plates, we observed that the degree of the mating defect of Δ*hos2* mutant might be dependent on nutritional conditions. This result will be further discussed in the manuscript (see Discussion). The other HDAC mutants mated normally (S5 Fig and [35]).

To determine whether Hos2 contributes to cell fusion, we also analysed the filamentation of wild-type and Δ*hos2* mutants in the solopathogenic strain SG200. This strain contains genes encoding a compatible bE1/bW2 heterodimer, as well as a gene constitutively expressing the opposite mating type pheromone (*mfa2*) [40]. This allows haploid SG200 cells to form filaments and infect maize plants without needing to mate with a compatible partner. We found that the deletion of *hos2* in this background severely impaired the formation of white, fuzzy colonies (Fig 2D), indicating that Hos2 has a post-fusion mating role. We then verified the virulence capacity of the SG200Δ*hos2* mutant and observed that Hos2 is also required for full pathogenicity in this genetic background (Fig 2E and 2F). Significantly, the virulence defects shown by SG200Δ*hos2* were comparable to those seen in FB1Δ*hos2* FB2Δ*hos2* mutant crosses, indicating that the post-fusion role of Hos2 is probably responsible for the reduced pathogenicity of Δ*hos2* mutant cells.

### Induction of *b* genes rescues Δ*hos2* filamentation defects

**Fig 3:**
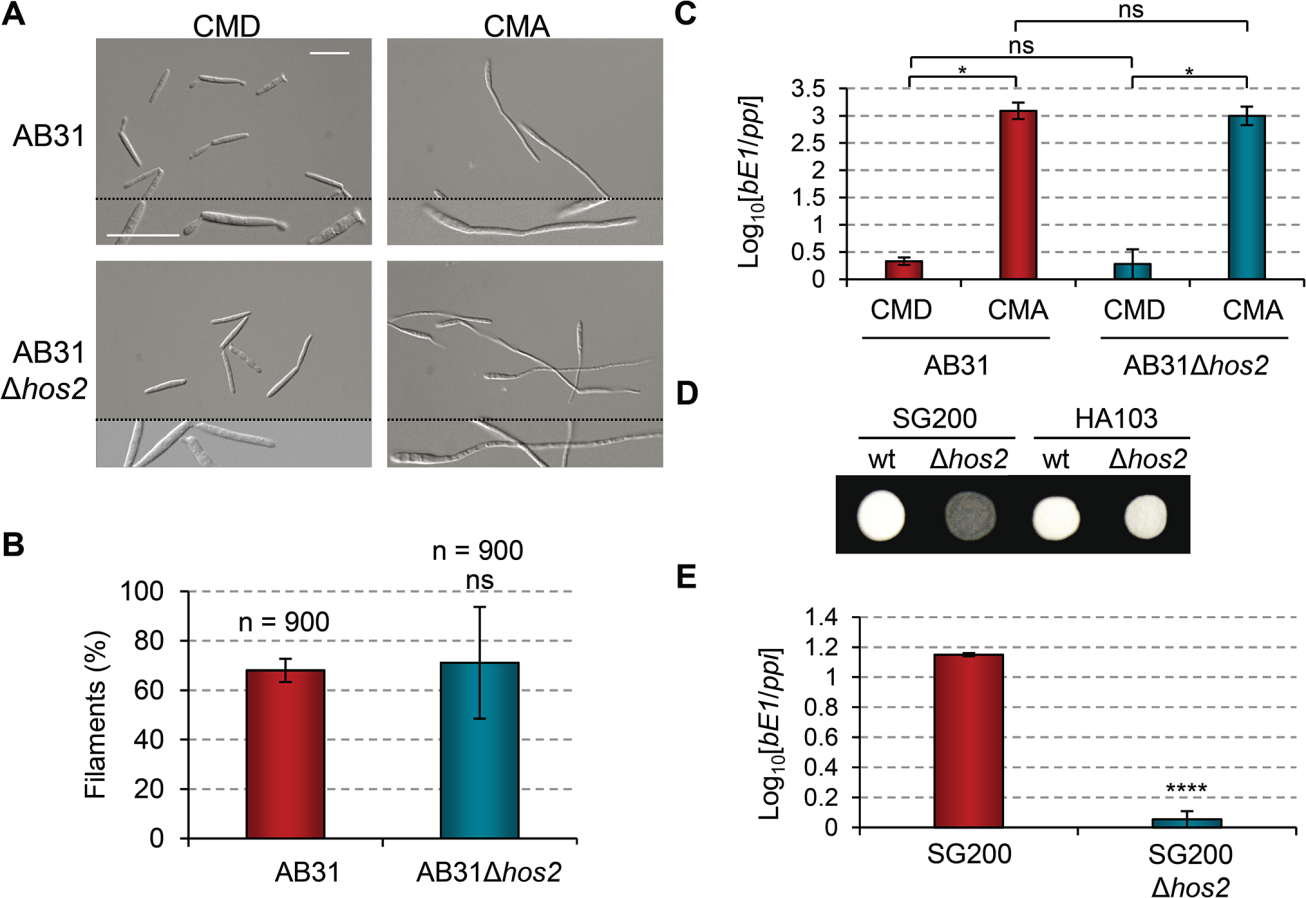
*b* gene induction rescues Δ*hos2* filamentation defects. (A) The filament forming capacity of AB31 and AB31Δ*hos2* strains. Expression of a compatible bE/bW heterodimer under the control of the *crg1* promoter was induced by shifting from glucose to arabinose containing CM media. Filaments were observed 8 hours after induction. (B) Quantification of the filamentation phenotype of the indicated strains 8 hours after *b* gene induction. Mean values and SDs from three independent experiments are shown. The total number of filaments counted is indicated above each column. *ns* denotes not statistically significant difference (t-test, *p*>0.05) (C) *bE1* expression levels, relative to the constitutively expressed *ppi* gene, in AB31 and AB31Δ*hos2* strains growing in CMD or CMA 8 hours post-induction. Mean values and SDs from three independent experiments, each containing three technical replicates, are shown. Relative values are shown normalised to one of the biological replicates of the sample with the lowest expression level (AB31/CMD in this case) that is assigned a value of 1. Statistically significant (*) and not significant (*ns*) differences are shown (Duncan’s new multiple range test, *p*<0.05) (D) Constitutive expression of *b* genes restores the filamentation defect of SG200Δ*hos2* mutants. The filamentation phenotypes of HA103 and HA103Δ*hos2* strains grown on PD charcoal plates for 24h. SG200 and SG200Δ*hos2* strains were used as controls. (E) *bE1* expression levels in SG200 and SG200Δ*hos2* strains grown on CM charcoal plates for 24 hours. Mean *bE1* expression values relative to *ppi* and SDs from three independent experiments, each containing three technical replicates, are shown. Values are normalised to one of the biological replicates of the sample with the lowest expression value (SG200Δ*hos2*) that is assigned a value of 1. *t*-test retrieved a statistically significant difference in *bE1* expression level between SG200 and SG200Δ*hos2*, with *p*<0.0001 (denoted by ****).

In *U*. *maydis*, post-fusion filament formation is controlled by *b* mating-type genes, which are necessary and sufficient to induce filamentation and pathogenicity [40]. Upon formation of the bE/bW heterodimer, several transcription factors are activated, some of which are known to be essential for filament formation and virulence [41–43]. To check for a genetic interaction between *hos2* and *b* genes, we took advantage of the AB31 strain, which contains a compatible *b* heterodimer under the control of the arabinose inducible promoter, *crg1* [41]. This strain can either divide by budding, when grown on complete media containing glucose as the sole carbon source, or switch to filamentous growth on complete media containing arabinose. Deletion of *hos2* in the AB31 background did not affect filamentation, as compared to a wild-type control (Fig 3A and 3B). We verified that *b* gene expression was comparable between Δ*hos2* and the wild-type strains (Fig 3C). Similar results were obtained when *hos2* was deleted in a HA103 genetic background (Fig 3D), which allows the constitutive expression of a compatible *b* heterodimer [18]. Thus, the constitutive expression of *b* genes restores the reduced filamentation observed in the SG200Δ*hos2* strain. These results suggest that Hos2 controls post-fusion filamentation upstream of the *b* locus.

We hypothesised that either Hos2 is required for *b* gene expression, or that it controls filament formation via an independent pathway that can be compensated for by the induction of *b* genes. To check the expression level of *b* genes in Δ*hos2* mutants, we grew cells on complete charcoal-containing medium, and analysed *b* gene expression levels by RT-qPCR. As shown in Fig 3E, *bE1* mRNA levels were reduced in Δ*hos2* mutants, suggesting that Hos2 is required for the expression of *b* genes.

### Hos2 is required for pheromone-induced conjugation tube formation

**Fig 4:**
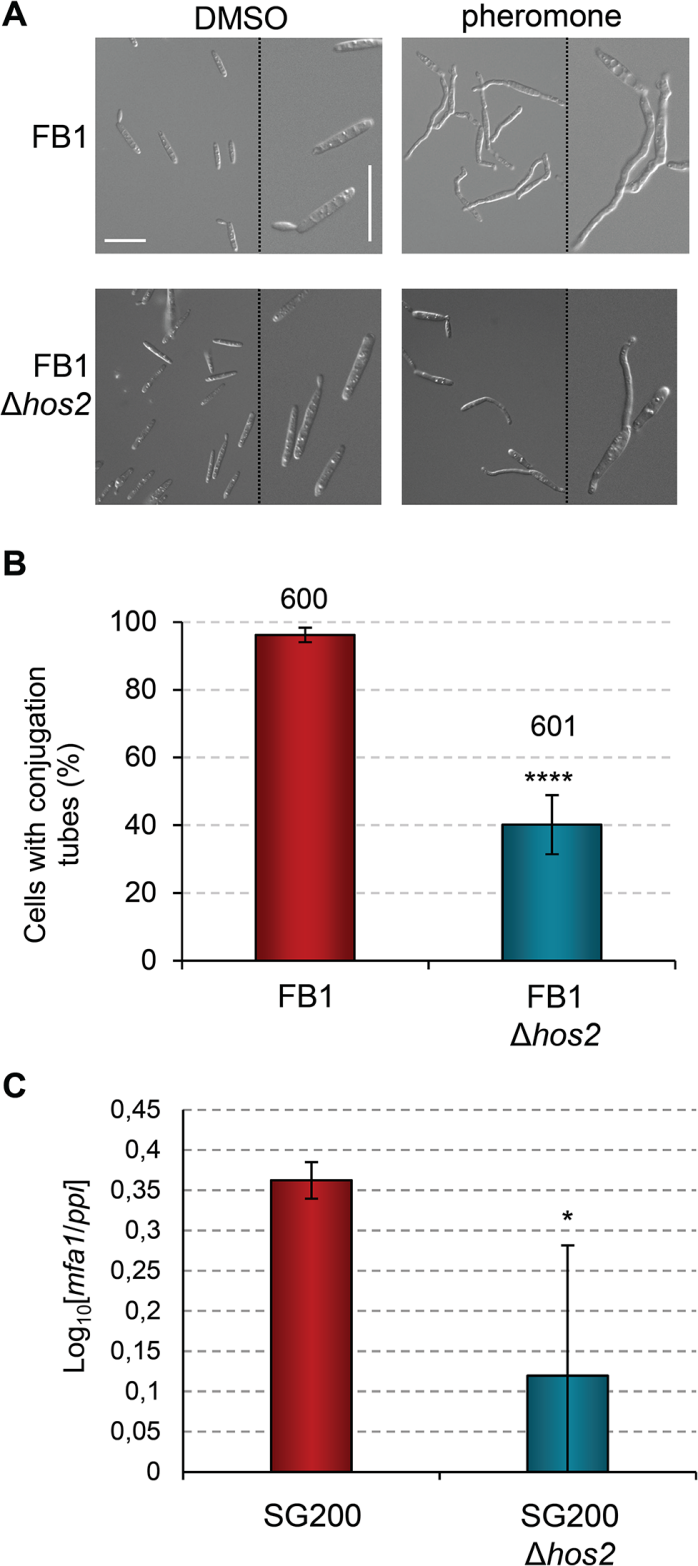
Hos2 is required for conjugation tube formation upon pheromone stimulation. (A) Representative image of conjugation tube formation upon a2 pheromone stimulation in FB1 and FB1Δ*hos2* strains. Images were taken 5 hours post-pheromone addition. The pheromone solvent, DMSO, was used instead of pheromone as a negative control. (B) Quantification of the pheromone defective phenotype of Δ*hos2* cells 5 hours post-pheromone stimulation. Mean values and SDs from three independent experiments are shown. The total number of cells counted is indicated above each column. t-test retrieved a statistically significant difference in conjugation tube formation between the FB1 and FB1Δ*hos2*, with *p*<0.0001 (showed as ****). (C) *mfa1* expression levels relative to *ppi*. Three independent experiments, each with three technical replicates were performed. Mean values and SDs for these experiments are shown. Values are normalised to one of the biological replicates of the sample with the lowest expression level (SG200Δ*hos2*) that is assigned a value of 1. An asterisk denotes a statistically significant difference with a *p* < 0.05 (t-test).

We have shown that Hos2 plays an important role in post-fusion filamentation upstream of *b* genes. Considering that the direct regulator of *b* genes, Prf1, is also responsible for the expression of the pheromone and receptor genes, we wondered whether Hos2 could also be affecting pre-fusion events during mating. To do this, we performed a pheromone stimulation assay by exposing wild-type and Δ*hos2* cells to synthetic a2 pheromone, then checking conjugation tube formation 5 hours post-pheromone addition. As shown in Fig 4A and 4B, the *hos2* mutant showed a 50% reduction in conjugation tube formation upon pheromone stimulation. This result indicates that Hos2 has a dual function in *U*. *maydis* dimorphism by controlling pre- and post-fusion mating events. Accordingly, the expression of the gene encoding the Mfa1 pheromone, *mfa1*, was reduced in *hos2* mutants compared to wild-type cells (Fig 4C).

Interestingly, addition of the HDAC inhibitor Trichostatin A (TSA), phenocopied the Δ*hos2* mutant conjugation tube formation defect. Incubation of wild-type FB1 cells with TSA prior to pheromone addition led to a decrease in the number of cells able to develop conjugation tubes (S6A Fig), without affecting cell viability (S6B Fig). These results suggest that the chemical inhibition of Hos2 activity recapitulates the mating phenotypes observed in Δ*hos2* strains. Furthermore, overexpression of *hos2* under the *otef* promoter at the *ip* locus, restored the filamentation capacity of SG200Δ*hos2* (S7A and S7B Fig). We verified that overexpression of *hos2* did not cause any mating, filamentation or virulence phenotype, by integrating the same construct in the FB1 or SG200 wild-type strains (S7B–S7D Fig). Altogether, these results suggest that the observed phenotypes are a consequence of the loss of *hos2*.

### Hos2 is not downstream of the pheromone responsive Fuz7 MAPK cascade

**Fig 5:**
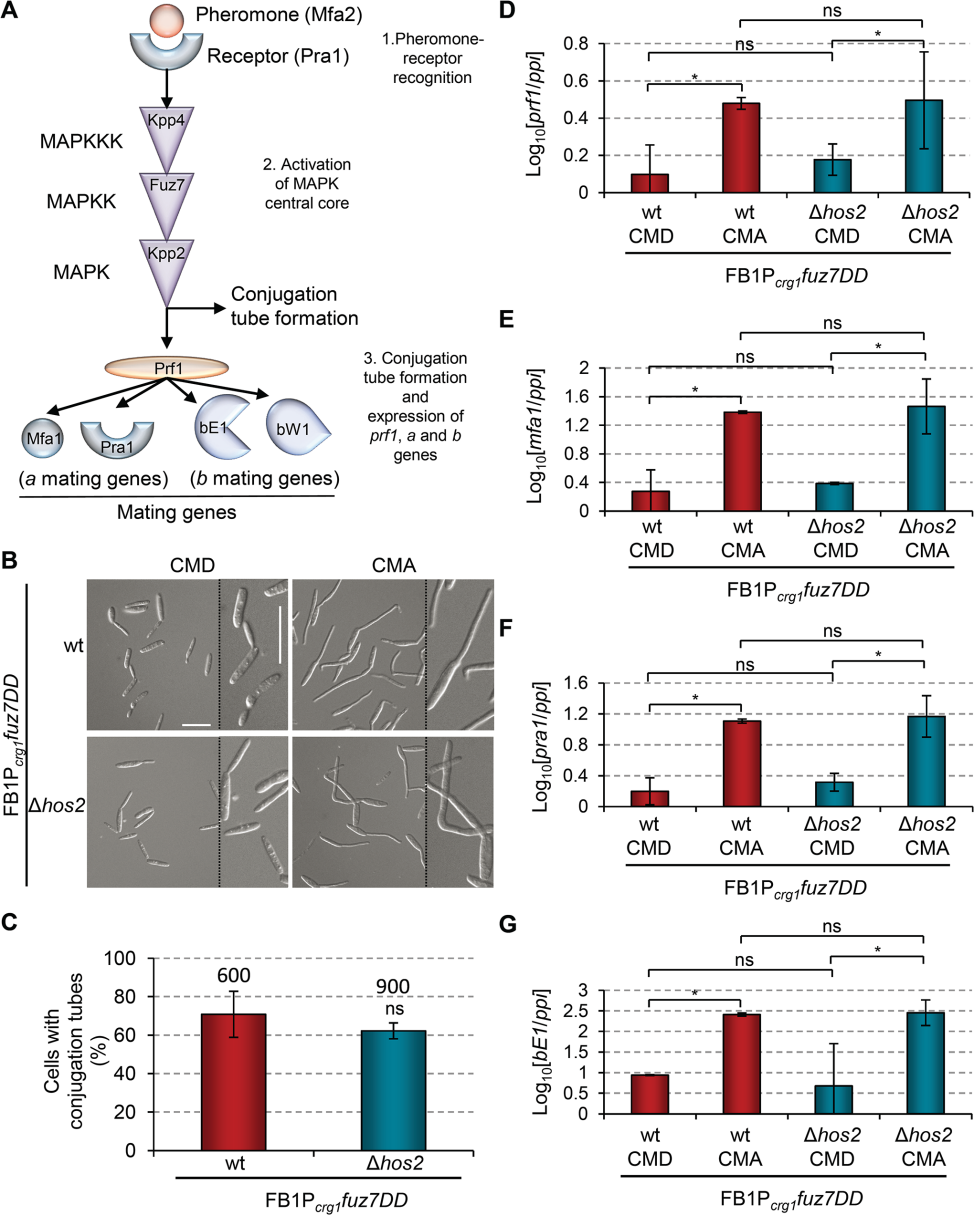
Hos2 is not downstream of the pheromone responsive Fuz7 MAPK cascade. (A) Schematic representation of the pheromone signalling pathway via the MAP kinase cascade. The events that following pheromone-receptor recognition are numbered. (B) Induced expression of the constitutively active Fuz7 MAPK kinase allele, *fuz7DD*, restores conjugation tube formation in Δ*hos2* mutants. *fuz7DD* induction was performed by shifting from glucose (non-inducing) to arabinose (inducing) containing CM media. Images shown were taken 5 hours after induction. (C) Quantification of *fuz7DD*-induced conjugation tube formation in the indicated strains 5 hours post-induction. Mean values and SDs from three independent experiments are shown. The total number of cells counted is indicated above each column. *ns* denotes not statistically significant differences (t-test, *p*>0.05) (D-G) Expression levels of the indicated genes, relative to *ppi*, in FB1*Pcrg1fuz7DD* and the corresponding *hos2* mutant in glucose and arabinose containing CM media, 5 hours post *fuz7DD* induction. Mean values and SDs from three independent experiments, each with three technical replicates, are shown. Values are normalised to one biological replicate of the sample with the lowest expression value, that is assigned a value of 1. One asterisk denotes *p*<0.05 (Duncan’s new multiple range test). *ns* denotes not statistically significant differences.

The role of Hos2 in pre- and post-fusion mating events, as well as in the expression of *a* and *b* genes, prompted us to examine whether Hos2 controls these processes in response to the pheromone responsive MAP kinase cascade. To test this possibility, we used the FB1P*crg1fuz7DD* strain which contains a constitutively active Fuz7 MAPK kinase allele under the control of the *crg1* promoter [22]. Expression of the constitutively active *fuz7DD* allele, by switching from glucose to arabinose containing media, induced the expression of both *prf1*, the *a* and *b* genes, and conjugation tube formation through an unknown pathway (Fig 5A; see also S1 Fig and [8] for details). As shown in Fig 5B and 5C, expression of the *fuz7DD* allele in a Δ*hos2* background promotes conjugation tube formation to a similar level to that observed in the wild-type control strain, indicating that Hos2 does not control conjugation tube formation downstream of the Fuz7 MAPK kinase. Moreover, the arabinose-dependent induction of *prf1*, *mfa1*, *pra1* and *bE1* was comparable between Δ*hos2* mutants and wild-type controls (Fig 5D–5G). *fuz7DD*-induced conjugation tube formation and mating type gene expression both require MAPK Kpp2 activity (Fig 5A and [22]); thus, our results strongly suggest that Hos2 does not function downstream of the MAPK cascade in the control of pre- and post-fusion events during mating.

### Hos2 controls virulence independently of Tup1

**Fig 6:**
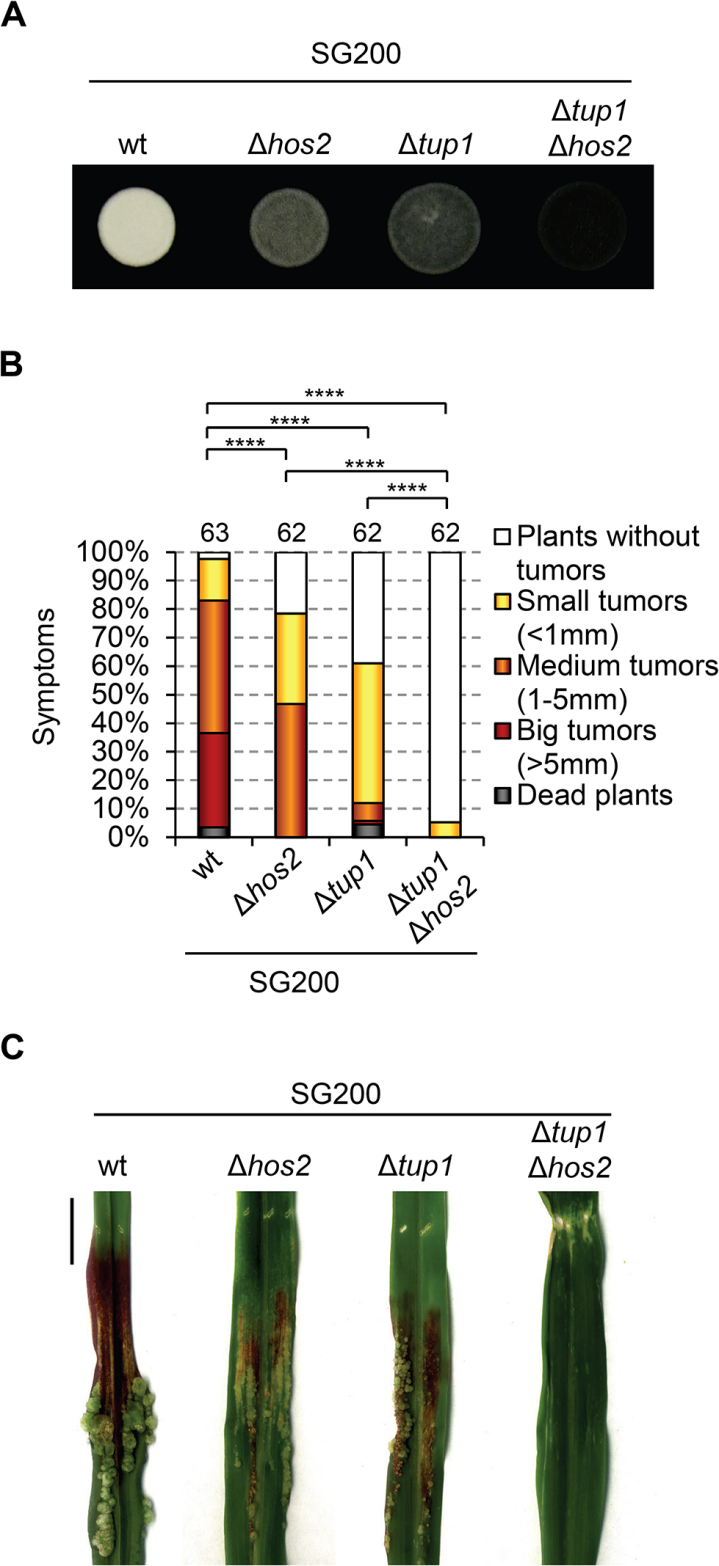
Hos2 controls virulence independently of Tup1. (A) Filamentation phenotypes of the SG200 strain and its derivative single and double mutants for Δ*hos2* and Δ*tup1* on PD charcoal plates. (B) Quantification of the virulence phenotype of the indicated strains 14 dpi. Mean values for three independent experiments are shown. The total number of infected plants is indicated above each column. **** means a statistical significant difference with *p*<0.0001 (Mann-Whitney test). (C) Tumours of maize plants induced by infections with the indicated strains 14 dpi. Scale bar = 1 cm.

If Hos2 is not downstream of the MAPK cascade, it could act in either an upstream or a parallel pathway. To address this question we turned to our previous work, which showed that Tup1 controls dimorphism and virulence in *U*. *maydis* [27], with many similarities to what we describe here for Hos2. Both proteins are involved in virulence by controlling pre- and post-fusion events during mating and, interestingly, Tup1 is known to control gene expression through interaction with histone deacetylases [44, 45]. However, unlike Hos2, Tup1 is required for *fuz7DD*-induced *prf1* and *a* and *b* gene expression (Fig 5, and [27]). Therefore, it is unlikely that Tup1 functionally interacts with Hos2 to control dimorphism and virulence. To confirm this, we decided to examine how Tup1 and Hos2 interact genetically. We constructed a double mutant SG200Δ*tup1*Δ*hos2* strain and analysed its virulence in maize plants. If Hos2 acts exclusively upstream of the MAPK pathway, we would expect an epistatic relationship with Tup1. As expected, double Δ*tup1*Δ*hos2* mutants were unable to form filaments on charcoal-containing plates, as is the case for either single mutant (Fig 6A). Interestingly, pathogenicity was severely reduced in Δ*tup1*Δ*hos2* mutants, compared to the wild-type or either of the single mutant strains (Fig 6B and 6C). These results indicate that both genes have independent regulatory roles during pathogenic development rather than acting together in the control of virulence genes. This interpretation is consistent with a putative role for Hos2 in the control of the dimorphic switch and virulence that is independent of the MAPK cascade.

### Hos2 is required for cAMP-induced expression of mating-type genes

**Fig 7:**
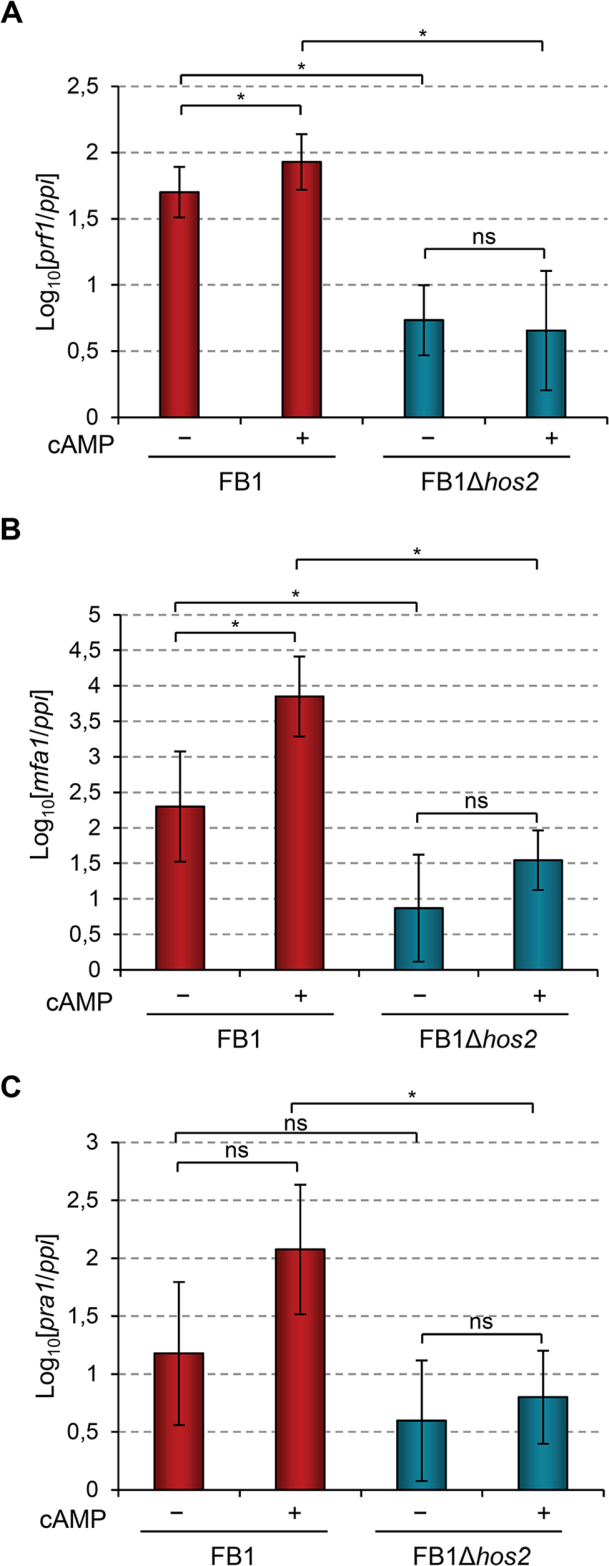
Hos2 is required for cAMP-induced expression of mating-type genes. (A-C) Expression level of the indicated genes relative to *ppi*. Strains were grown in PD broth for 8 hours with (+) or without (−) 6 mM cAMP. Mean values and SDs from three independent experiments, each containing three technical replicates, are shown. Values are normalised to one of the biological replicates of the sample with the lowest expression value that is assigned a value of 1. Statistically significant (*) and not significant (*ns*) differences are shown (Duncan’s new multiple range test, *p*<0.05).

In addition to the MAPK cascade, *U*. *maydis* dimorphism and mating-type gene expression are also controlled by the cAMP-PKA pathway (S1 Fig). Addition of exogenous cAMP to wild-type *U*. *maydis* cell cultures induces the expression of mating-type genes and cAMP pathway mutants affect this induction [19, 21, 23, 46–49]. Therefore, we looked at whether Hos2 might control the expression of mating-type genes in response to activation of the cAMP pathway. To do this, we measured the expression of *prf1*, *mfa1* and *pra1* in FB1Δ*hos2* cells growing in rich liquid medium (PD broth) with or without the addition of exogenous cAMP. As shown in Fig 7, the addition of cAMP was unable to induce the expression of mating- type genes in Δ*hos2* mutants. These results strengthen the hypothesis that Hos2 functions independently of the MAP kinase pathway to control mating-type gene induction. Additionally, even in the absence of cAMP, the expression of mating-type genes was reduced in Δ*hos2* mutants, suggesting that Hos2 is important for maintaining basal mating-type gene expression levels under these conditions.

### Hos2 binds to mating type genes to control their expression

In order to determine whether the effect of Hos2 on mating-type gene expression is direct, we performed chromatin immunoprecipitation (ChIP) assays followed by qPCR. For this purpose we constructed the strain FB1Hos2-HA3, in which the endogenous Hos2 protein is tagged with three copies of the HA epitope. This strain was able to form conjugation tubes upon pheromone stimulation in a comparable way to the wild-type control (S8 Fig), indicating that the Hos2-HA3 allele is functional.

**Fig 8:**
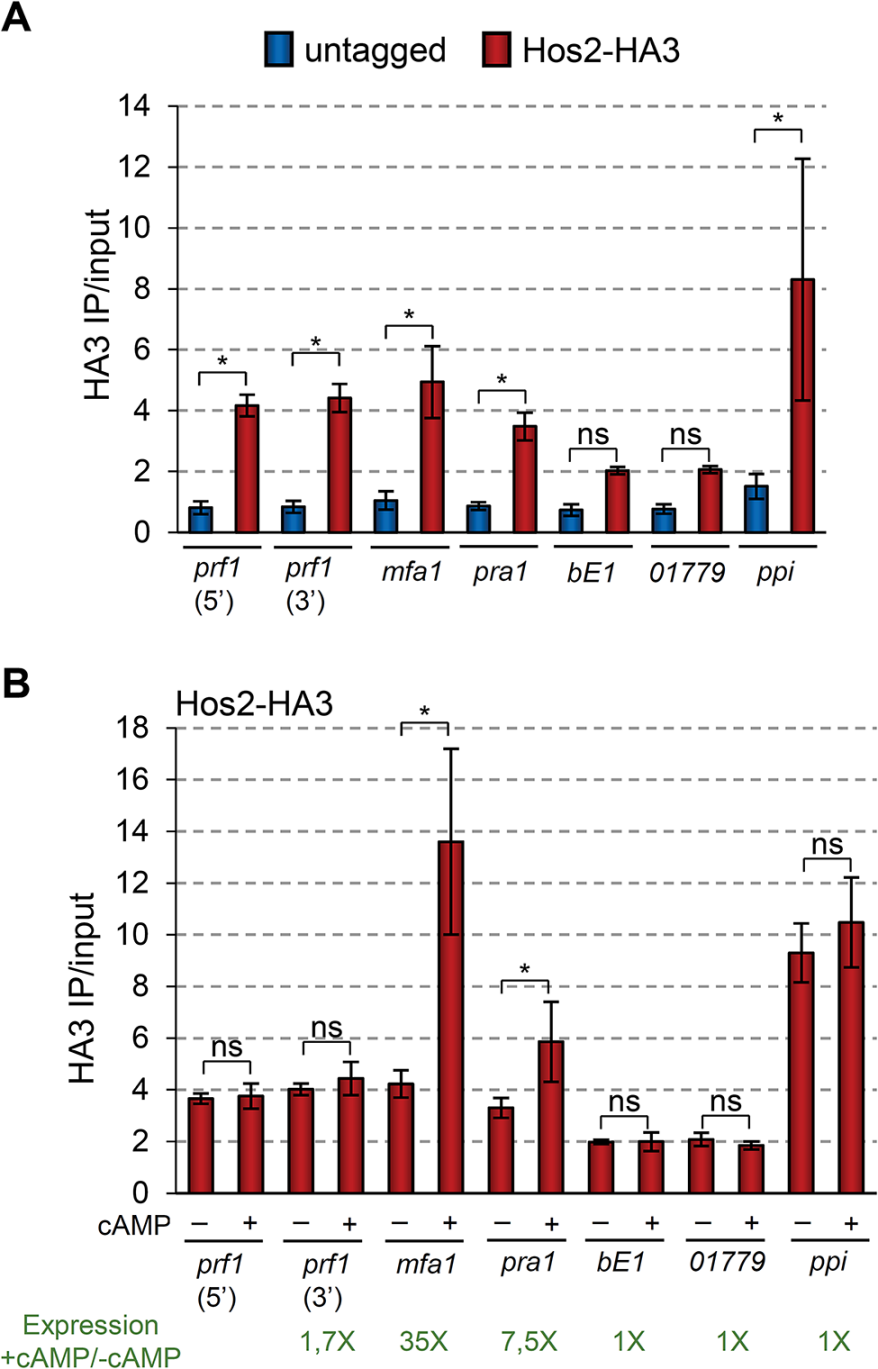
Hos2 directly binds to mating genes. (A) ChIP analysis using an anti-HA antibody on chromatin extracts from either an untagged strain or a Hos2-HA3 strain, grown in PD. Inmunoprecipitated DNA was analysed by qPCR, amplifying open reading frames or specific regions within the ORFs (5’ or 3’) indicated below the graph. Values correspond to the amount of DNA recovered in the HA IP divided by the amount of DNA in the corresponding input extract. Mean values and SDs from four independent experiments, each with three technical replicates are shown. Statistically significant binding of Hos2-HA3 (red) to each locus by comparison to the untagged (blue) strain is indicated with * (Duncan’s new multiple range test, *p*<0.05). All pairwise comparisons involving Hos2-HA3 at *bE1* locus were statistically significant (Duncan’s new multiple range test, *p*<0.05), except when compared to *pra1* or *01779*. The same was true for comparisons involving Hos2-HA3 at locus *01779*. (B) ChIP analysis was performed and analysed as in (A), except that strains were grown in PD with or without the addition of 6 mM cAMP for 8 hours. For simplicity, values for the untagged strains are not shown, but were identical to those shown in A and did not vary upon cAMP addition. Statistically significant differences regarding the effect of cAMP addition in Hos2 binding to each locus are denoted with * (Duncan’s new multiple range test, *p*<0.05). Numbers in green indicate the fold expression increase in of the corresponding gene upon cAMP addition, as measured by RT-qPCR and shown in Fig 7 (note the Log_10_ in *y* axis of Fig 7).

In *S*. *cerevisiae* and *C*. *albicans* Hos2 is recruited to gene bodies in a transcription dependent manner [29, 50]. Thus, we decided to check whether *U*. *maydis* Hos2 binds to the gene bodies of mating-type genes. As shown in Fig 8A, there was a significant enrichment of Hos2-HA3 within the *prf1*, *mfa1* and *pra1* open reading frames (ORFs), as compared to the HA ChIP signal obtained in an untagged strain. As a negative control, we analysed the enrichment of Hos2 at the appressorium specific *um01779* gene, which is expressed only upon induction of appressoria formation [12]. At this gene, Hos2-HA3 binding was not significant compared to the untagged control. A similar result was obtained for Hos2-HA3 associated with the *bE1* ORF. In conclusion, our ChIP experiments reveal that Hos2 directly binds to *prf1*, *mfa1* and *pra1* mating-type gene sequences, where it most likely acts to control their expression.

ChIP-seq experiments in *S*. *cerevisiae* and *C*. *albicans*, clearly show that Hos2 occupancy positively correlates with gene expression levels [29, 50]. Therefore, we analysed how Hos2 binding to mating-type genes changes upon cAMP addition, which induces their expression (Fig 7). We found that the addition of cAMP caused a significant increase in Hos2 binding to *mfa1* and *pra1* ORFs (Fig 8B). Increased Hos2 binding to *mfa1* and *pra1* correlated well with their increased expression upon cAMP induction (Fig 7). Hos2 *prf1* binding was unaffected by cAMP addition (Fig 8B), consistent with its modest induction (Fig 7). As a control, we then tested the cAMP-induced recruitment of Hos2 to three different loci. Hos2 binding remained not significant at the *bE1* gene, whose expression does not respond to cAMP addition [21]. A similar result was observed at *um01779*, whose expression is also cAMP independent [12]. Finally, as a positive control, we detected significant enrichment of Hos2 at the strongly constitutively-expressed *ppi* gene, irrespective of cAMP addition. Thus, we conclude that Hos2 directly regulates the expression of mating-type genes in *U*. *maydis* in response to cAMP signalling. This is likely to account for the expression and mating defects observed in *hos2* mutants.

### *U*. *maydis* Rpd3 homologs, Hda1 and Hda2, do not act redundantly with Hos2 to control the dimorphic switch or virulence

**Fig 9:**
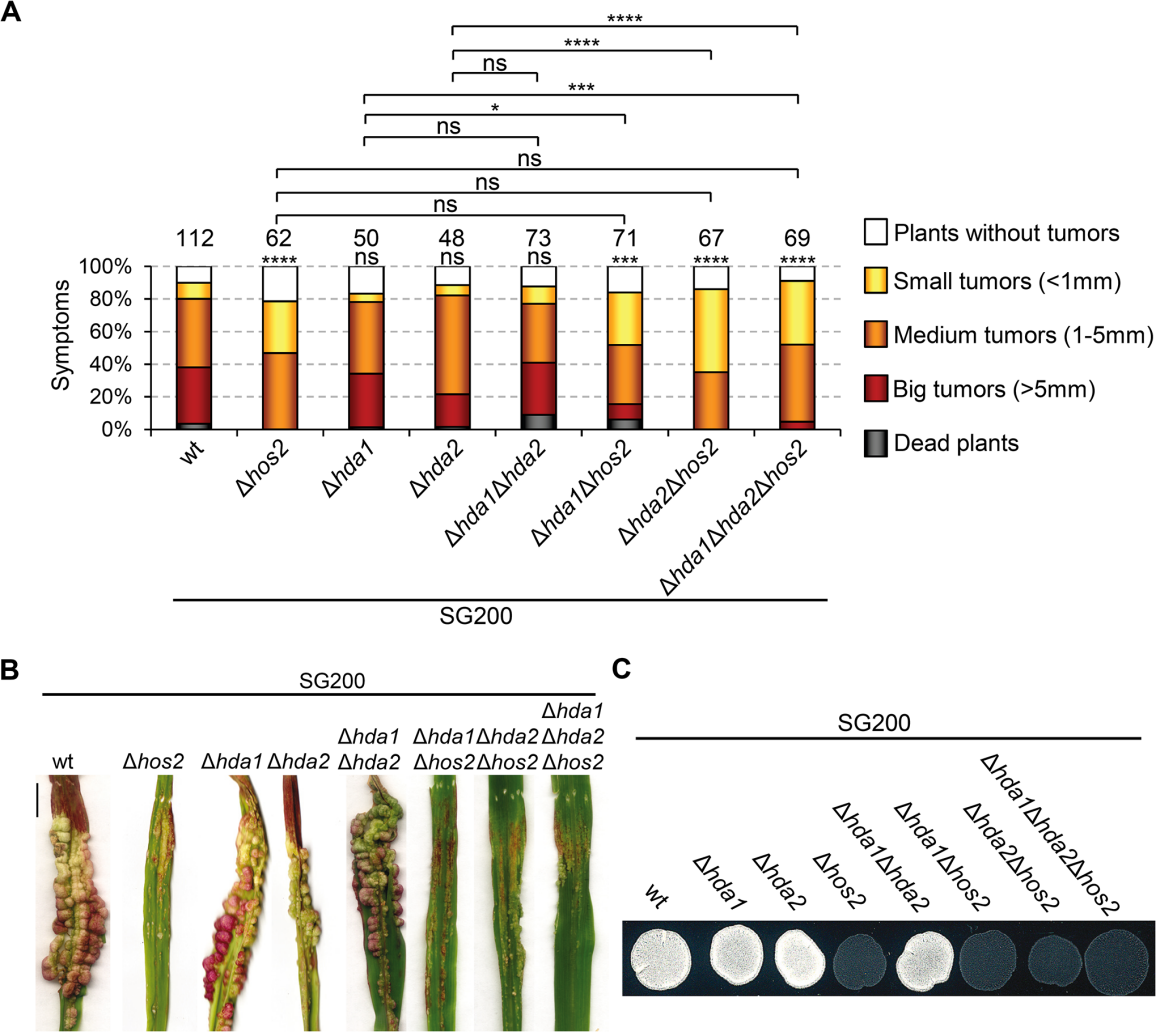
Hda1 and Hda2, are not redundant with Hos2 in the control of dimorphism and virulence. (A) Quantification of infection symptoms caused by the indicated strains on maize plants 14 dpi. Mean values of three independent experiments are shown. Total number of infected plants are indicated above each column. Statistically significant differences are indicated with asterisks (Mann-Whitney test; the number of asterisks are used for the following *p*-values: * for p<0.05, ** for p<0.01, *** for p<0.001 and **** for p<0.0001. Asterisks placed above each column and bellow the total number of infected plants correspond to comparisons with the SG200 wild-type strain. (B) Representative image of tumours induced by wild-type and *hos2* mutant strains 14 dpi. Scale bar = 1 cm. (C) Filamentation on PD charcoal plates of the indicated strains after 48 hours incubation at 25°C.

HDACs have redundant roles in several organisms [51–53]. As described in Fig 1A, *U*. *maydis* has two putative *S*. *cerevisiae* Rpd3 orthologues. Examination of the double Δ*hda1*Δ*hda2* mutant phenotypes in a SG200 background, revealed that neither maize plant virulence nor filament formation on charcoal plates were significantly affected (Fig 9). This indicates that the two putative *U*. *maydis* Rpd3 orthologues do not act redundantly in these processes.

In *S*. *cerevisiae*, ScRpd3 and ScHos2 act redundantly to control *FLO11*, a gene involved in budding yeast dimorphism [53]. Therefore, we tested whether a similar genetic interaction might control pathogenicity in *U*. *maydis*. We constructed all possible double and triple mutant combinations in a SG200 genetic background and examined their virulence and filamentation phenotypes. All combinations showed phenotypes comparable to those observed in single Δ*hos2* mutants (Fig 9). These observations confirm that Hda1 and Hda2 do not control corn smut fungus pathogenic development. Furthermore, our data suggest that there are no significant functional redundancies between the ScRpd3 homologs, Hda1 and Hda2, and Hos2 in the control of dimorphism and virulence in *U*. *maydis*.

### Hos2 and Clr3 genetically interact in the control of virulence in *U*. *maydis*

**Fig 10:**
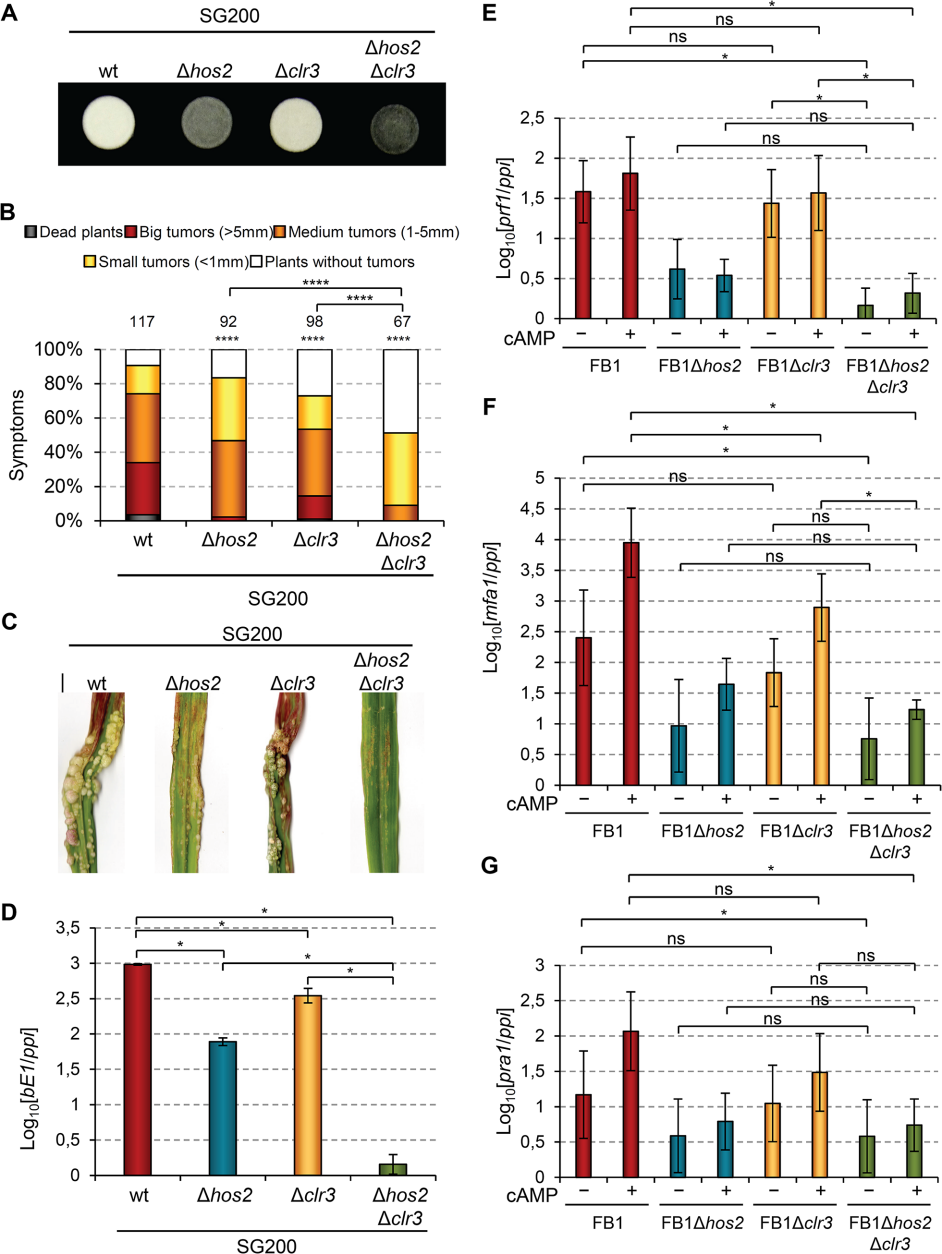
Hos2 and Clr3 genetically interact in the control of virulence. (A) Filamentation on PD charcoal plates of SG200 and its derivative mutants for *hos2* and *clr3* growing for 24 hours at 25°C. (B) Quantification of the virulence capacity of the indicated strains on maize plants 14 dpi. Mean values of three independent experiments are shown. Total number of infected plants are indicated above each column. All pairwise comparisons are statistically significant (**** denotes *p*<0.0001, Mann-Whitney test), except when comparing Δ*hos2* with Δ*clr3* single mutants. (C) Images of tumours induced by the indicated strains 14 dpi. (D) Expression of *bE1* relative to *ppi* in the indicated strains. Mean values and SDs from three independent experiments, each containing three technical replicates, are shown. Values are normalised to the sample with the lowest expression value (SG200Δ*hos2*Δ*clr3*) that is assigned a value of 1. Data for wild-type and Δ*hos2* mutants are shown in Fig 3. (E-G) Expression level of the indicated genes relative to *ppi*. Mean values and SDs from three independent experiments, each containing three technical replicates, are shown. Values are normalised to one biological replicate of the sample with the lowest expression value (FB1Δ*hos2*Δ*clr3* without cAMP) that is assigned a value of 1. Statistically significant (*) or not significant (*ns*) differences for pairwise comparisons involving *clr3* and *hos2*-*clr3* genetic interaction are indicated (Duncan’s multiple range test, *p*<0.05). Data and statistical analysis for wild-type and Δ*hos2* mutant are shown in Fig 7.

In the human pathogen *C*. *albicans*, CaHos2 and CaHda1 have been shown to play opposing roles in the control of morphological changes [54]. Thus, we wondered whether an analogous genetic interaction might occur during the *U*. *maydis* yeast to hypha transition. To test this, we deleted the *U*. *maydis clr3* gene (Ca*hda1*) in the SG200 background, and examined its filamentation capacity alone or in combination with Δ*hos2*. As shown in Fig 10A, *clr3* deletion did not rescue the filamentation defects of Δ*hos2* mutants, suggesting that Clr3 does not antagonise Hos2 control of the *U*. *maydis* dimorphic switch. As with FB1Δ*clr3* FB2Δ*clr3* mutant crosses (Fig 1B), we detected a decrease in SG200Δ*clr3* mutant virulence capacity (Fig 10B and 10C). Furthermore, we observed an even stronger virulence reduction in the SG200Δ*hos2*Δ*clr3* double mutants, versus either single mutant. Altogether, these results show that Clr3 contributes to *U*. *maydis* pathogenicity, particularly when Hos2 is absent, and indicate that Hos2 and Clr3 play either redundant or independent functions during fungal virulence.

To further characterise this genetic interaction, we measured the expression levels of *b* mating-type genes in Δ*clr3* single and Δ*hos2*Δ*clr3* double mutants during filamentation on charcoal-containing medium. As shown in Fig 10D, Δ*clr3* mutants showed a mild reduction in *b* gene expression, but retaining sufficient expression to promote filamentation (Fig 10A). Interestingly, Δ*hos2*Δ*clr3* double mutant showed lower *b* gene expression than either Δ*clr3* or Δ*hos2* single mutants. We also measured the expression of *a* mating-type genes and *prf1* in response to addition of exogenous cAMP. Similarly to what we found for *bE1* gene, we observed a mild reduction of *mfa1* expression in *clr3* mutants upon cAMP addition (Fig 10E–10G). However, we did not observe a synergistic reduction in the expression of this gene in Δ*hos2*Δ*clr3* double mutant. Overall, these observations indicate that Hos2 and Clr3 participate in the control of mating-type gene expression through the cAMP pathway. Our results also suggest some redundancy between Hos2 and Clr3, at least in the regulation of *b* gene expression.

## Discussion

Morphological changes are critical for pathogenic fungi to be able to infect their hosts and, consequently, are tightly controlled. Here, we report a comprehensive characterisation of the roles of HDACs over the life cycle of the plant pathogenic fungus *U*. *maydis*. We observed that of all the HDACs only Hos2 and, to a lesser extent, Clr3, have significant roles in cell growth, morphology, dimorphism or virulence. Moreover, we identified the signalling pathways that regulate Hos2 and Clr3 to control the yeast-hypha transition in this fungus. Finally, we described putative functional redundancies between Hos2 and Clr3 in the control of *U*. *maydis* pathogenicity.

Hos2 plays crucial roles during fungal pathogenesis [32–34]. In the plant pathogenic fungi *Magnaporthe oryzae*, *Cochliobolus carbonum* and *Fusarium graminearum*, loss of Hos2 compromises appressorium biology during the plant penetration and post-penetration stages of pathogenic development [33, 34, 55, 56]. Our results reveal another fungus in which Hos2 is required for virulence. However, our study suggests that Hos2 controls pathogenicity by a different mechanism to the ones described for other plant pathogenic fungi. Indeed, the reduced virulence of *U*. *maydis* Δ*hos2* mutants appears to be due to their inability to shift from yeast-like to polarised growth, which is needed to form infective filaments. Interestingly, yeast-to-hypha transition defects have also been noticed in Δ*hos2* mutants of the human pathogen *C*. *albicans*. However, in this fungus, *hos2* deletion results in constitutive filamentation, which is the opposite phenotype to the one we have observed in *U*. *maydis* [33].

Strikingly, the roles we have identified for Hos2 in this work have a number of parallels with our previous findings, including opposing regulatory roles between *U*. *maydis* and *C*. *albicans*, for the general transcription repressor Tup1. Our results show that Δ*hos2* mutants are impaired at pre- and post-fusion events during mating, again resembling what we had previously observed for Tup1 [27]. Interestingly, one of the mechanisms through which Tup1 controls gene expression is via interaction with histone deacetylases [44, 45]. One possibility is that Hos2 could be the HDAC recruited and controlled by Tup1 in its regulation of mating-type gene expression. However, a double Δ*tup1*Δ*hos2* mutant strain was almost non-virulent, showing a much more severe phenotype than the single mutants (Fig 6), suggesting independent roles for the two proteins. Consistent with this idea, Tup1 controls the expression of mating-type genes downstream of the pheromone responsive MAP kinase cascade [27], whereas we did not observe such an effect in Δ*hos2* mutants. Although the contribution of HDACs to Tup1 function in transcription initiation is well established, there is also evidence to suggest that Tup1 can regulate gene expression by HDAC-independent mechanisms. For example, Wong and Struhl, as well as Parnell and Stillman, showed that the repressive role of Tup1 in certain promoters relies mainly on its interaction with histone acetyl-transferase (HAT) complexes [57, 58]. Interestingly, it has recently been shown that deletion of the Gcn5 histone acetyl-transferase causes a constitutive filamentous phenotype in *U*. *maydis* [59, 60]. From this evidence we could propose a model whereby Tup1 controls the dimorphic switch by regulating the Gcn5 HAT, with the Hos2 HDAC regulated by another parallel mechanism.

By which mechanism does Hos2 control the dimorphic switch? One possibility is that *hos2* expression is controlled by the environmental signals that ultimately activate this transition. As shown in S9 Fig, *hos2* expression did not vary substantially in different media, upon activation of the cAMP or MAPK signalling pathways, or during filamentous growth in charcoal-containing media. These observations suggest that external cues regulate mating-type genes via changes to Hos2 activity rather than expression. Supporting this conclusion, we observed that the mating-type defects of Δ*hos2* mutants were more pronounced on charcoal-containing CM plates than on PD ones (Fig 2C). Thus, Hos2 might control mating in response to the nutrients present in the medium.

Our functional analyses of the MAPK cascade and of the cAMP-PKA pathway, reveal that Hos2 controls mating-type gene expression downstream of cAMP signalling. The simplest and most obvious explanation for this observation is that Hos2 directly targets mating-type genes in response to cAMP signalling. Our ChIP analysis confirms that Hos2 directly binds the *prf1* ORF, as well as some of its target genes. Furthermore, Hos2 recruitment responds to cAMP and positively correlates with the expression level of *mfa1* and *pra1* mating-type genes. Interestingly, Hos2 binding at *prf1* ORF does not respond to cAMP addition, suggesting that there might be differences in how Hos2 is recruited at *mfa1* and *pra1* on one hand or at *prf1* on the other hand. Altogether, it is likely that Hos2-mediated deacetylation of these genes promotes transcriptional elongation, as has been proposed for *S*. *pombe*, *S*. *cerevisiae* and *C*. *albicans* Hos2 target genes [37, 50, 61].

Strikingly, we detected even stronger Hos2 binding to the *prf1* promoter than along its ORF (S10A Fig). Interestingly, the *prf1* upstream regulatory region is particularly long, extending over 2 Kb. The strongest region of Hos2 binding overlapped with a cAMP- and nutrient-responsive upstream activating sequence (UAS) [19]. Upon cAMP addition, Hos2 binding slightly decreased specifically at this UAS (S10B Fig). This strong binding of Hos2 upstream of the *prf1* ORF was unexpected because *S*. *cerevisiae* and *C*. *albicans* Hos2 have been shown to mainly bind gene bodies [29, 31, 50]. An exciting possibility is that *U*. *maydis* Hos2 binds to this UAS to control the transcription of a putative long non-coding RNA, similar to what has been observed upstream of the *S*. *cerevisiae IME1* gene [62]. This could open up an exciting novel line of research toward understanding *prf1* expression regulation, which is critical for the dimorphic switch and *U*. *maydis* virulence.

Another non-mutually exclusive possibility is that Hos2 controls the activity of PKA, or its regulatory subunits, or affect the amount of intracellular cAMP. However, Δ*hos2* mutants showed sensitivity to TSA, accompanied by increased H4K16 acetylation (S3 Fig). Additionally, our ChIP experiments show that mating-type genes are direct targets of Hos2 (Figs 8 and S10). Altogether, our data strongly suggest that Hos2 directly regulates mating-type gene expression to control mating and virulence in *U*. *maydis*.

Interestingly, a previous study has indeed reported that the Hos2/Set3 complex functions in the *C*. *albicans* cAMP pathway. *C*. *albicans* mutants lacking the Hos2/Set3 complex are hyper-sensitive to filamentation-inducing signals [32]. This is in marked contrast with the role of *U*. *maydis* Hos2 that we have reported here. Interestingly, the effect of cAMP-PKA signalling is also different between *C*. *albicans* and *U*. *maydis*. Hyperactivation of this pathway in *U*. *maydis* causes a multiple budding phenotype, while its inhibition causes filamentation, as observed in mutations affecting the regulatory and catalytic subunits of PKA [47, 48]. In contrast, hyperactivation of the same pathway in *C*. *albicans* leads to filamentation. To conclude, the role of Hos2 in the cAMP pathway may be conserved between *C*. *albicans* and *U*. *maydis*, even though the pathway has opposite effects on dimorphism in the two organisms.

Although Δ*hos2* mutants clearly showed the most prominent virulence phenotype, we also noticed a modest infection defect in Δ*clr3* mutants. It is known that HDACs can compensate for each other in the regulation of gene expression [51–53]. Therefore, we wanted to check for functional redundancies between HDACs in the control of fungal virulence. In *C*. *albicans*, CaHos2 and CaHda1 (Clr3 in *U*. *maydis* and *S*. *pombe*) play opposite roles in the control of morphological changes [54]; however, how these two HDACs interact with each other to control dimorphism is still unknown. Interestingly, in *U*. *maydis*, Δ*hos2*Δ*clr3* double mutants exhibited much more severe pathogenesis defects than either of the single mutants. From these data we can conclude that Clr3 participates in *U*. *maydis* virulence, particularly when Hos2 is absent, revealing a genetic interaction between both genes during *U*. *maydis* infection. Like Hos2, we found that Clr3 is required for normal expression of *a* and *b* mating-type genes. Our analysis of filamentation, virulence and mating-type gene expression in single and double mutants suggests that Hos2 can compensate for Clr3 in *U*. *maydis*, in contrast to their opposing roles in *C*. *albicans* [54]. Nevertheless, a common role for Hos2 and Clr3 in the control of *C*. *albicans* dimorphism might be taking place too. Recently, CaHda1 has been shown to be required for the maintenance of filamentation in nutrient-poor media that lacks the preferred nitrogen source glutamine [63]. Interestingly, hyperfilamentation of *C*. *albicans* Δ*hos2* mutants occurs virtually in all conditions except for nitrogen starvation conditions, where they are unable to form filaments, while the wild-type can [54]. Thus, Hos2 and Clr3 might have a common role in the control of *C*. *albicans* dimorphism with respect to certain nutritional conditions.

In summary, we have shown that the class I histone deacetylase Hos2 has a crucial role in the control of dimorphism and virulence in *U*. *maydis*. Our data demonstrate that Hos2 binds to and is required for the expression of mating-type genes upon activation of the cAMP pathway. We believe that our results contribute to a better understanding of how HDACs regulate morphological changes and fungal pathogenic development. In the future, it will be interesting to study other HDAC complex components and which histone acetyltransferase is involved in these processes. Our results also provide interesting insights into the regulatory mechanisms governing the expression of virulence genes via conserved signalling pathways, which may be highly relevant to other fungal pathogens.

## Methods

### Strains and growth conditions

*Escherichia coli* DH5α was used for cloning purposes. Growth conditions for *E*. *coli* [64] and *U*. *maydis* [16, 65] have been described previously. *U*. *maydis* strains relevant to this study are listed in S1 Table.

For cell width and length analyses, exponentially growing cultures were generated by growing cells in YEPSL liquid media for 12 hours, diluting them in the same media to OD600 = 0.05 and then allowing them to reach OD600 = 0.8–1 prior to light microscopy examination. For the estimation of growth rates, *U*. *maydis* cells were grown until exponential phase in YEPSL at 28°C, diluted in the same media to OD600 = 0.05 and the number of cells counted. After eight hours of incubation at 28°C, cells were counted again and growth rates calculated. For cell viability assays, a total of 200 cells, grown in the conditions indicated in each case, were plated on YPD plates and incubated at 28°C for 3 days prior to colony counting.

For charcoal mating and filamentation assays, cells were grown on YEPSL until exponential phase, washed twice with sterile distilled water, spotted onto PD-charcoal plates and grown for 24–48 hours at 25°C. For RNA extractions, exponentially-growing cultured cells were spread out on CM charcoal plates at a concentration of OD600 = 0.1 per cm2.

Cells used to analyse the expression of *hos2* in different media were grown to OD600 = 0.5–0.8 for early exponential phase cultures (E) and to OD600 = 4 for late exponential phase cultures (L).

Induction of the *crg1* promoter in AB31 [41] or FB1P*crg1fuz7DD* [22] strains, and their derivatives, were done as previously described. Mating [16] and pheromone stimulation [22] assays were performed as previously described. To determine the effect of Trichostatin A (TSA; Sigma T8552) on conjugation tube formation, cells were grown in CMD until exponential phase (OD600~0.5–0.8); then 1 μg/ml of TSA was added to 1 ml of cell culture and incubated for 2 hours at room temperature prior to pheromone addition. Control cultures were treated only with DMSO or pheromone. In all cases, conjugation tube formation was quantified 5 hours after pheromone addition.

For pathogenicity assays, *U*. *maydis* strains were grown to exponential phase, concentrated to OD600 = 3, washed twice with sterile distilled water, and injected into 7 day old maize (*Zea mays*) seedlings (Early Golden Bantam). Tumour formation was quantified 14 days post infection.

For the cAMP-PKA pathway induction assays, cells were cultured to saturation in rich PD-broth media without cAMP, diluted in the same media and grown to exponential phase. Cells were then washed with sterile distilled water, diluted to OD600 = 0.2 and grown for 8 hours in PD-broth with 6 mM cAMP or the same media without cAMP. Cells were then recovered by centrifugation for 4’ at 4500 rpm and 4°C, washed twice with sterile distilled water and frozen in liquid nitrogen. RNA extraction was performed as described below for liquid cultured cells. RT-qPCR was performed as described below, using the corresponding primers (see S2 Table for sequences of primers).

### DNA and RNA procedures

Molecular biology techniques were used as previously described [64]. *U*. *maydis* DNA isolation and transformation procedures were carried out following the protocol of [1]. Deletion constructs were generated according to [66]. To generate single deletion *U*. *maydis* mutants for *hos1* (*um04234*), *hos2* (*um11828*), *hos3* (*um10914*), *hda1* (*um02065*), *hda2* (*um11308*) and *clr3* (*um2102*), fragments of the 5’ and 3’ flanks of their open reading frames were generated by PCR on *U*. *maydis* FB1 genomic DNA with the following primer combinations: UmHOS1KO5–1/UmHOS1KO5–2 and UmHOS1KO3–1/UmHOS1KO3–2; UmHOS2KO5–1/UmHOS2KO5–2 and UmHOS2KO3–1/UmHOS2KO3–2; UmHOS3KO5–1/UmHOS3KO5–2 and UmHOS3KO3–1/UmHOS3KO3–2; UmHDA1KO5–1/UmHDA1KO5–2 and UmHDA1KO3–1/UmHDA1KO3–2; UmHDA2KO5–1/UmHDA2KO5–2 and UmHDA2KO3–1/UmHDA2KO3–2; UmCLR3KO5–1/UmCLR3KO5–2 and UmCLR3KO3–1/UmCLR3KO3–2 (Sequences in S2 Table). These fragments were digested with *Sfi*I and ligated with the 1.9 Kb *Sfi*I carboxin resistance cassette, 2.7 Kb *Sfi*I hygromycin resistance cassette, or 1.5 Kb *Sfi*I neourseotricin resistance cassette as described previously [67]. Ligation products were then cloned into pGEM-T-EASY vector (Promega). Linear PCR-generated DNA was used for *U*. *maydis* transformation of each construct.

For *hos2* overexpression, the p123-*hos2* plasmid was generated. p123-*hos2* is a p123 [68] derivative whose eGFP fragment has been substituted with the *hos2* ORF. For this purpose, the *hos2* open reading frame was amplified by PCR with the oligonucleotides Umhos2ATGXmaISmaI and Umhos2StopNotI, containing *Xma*I and *Not*I restriction sites, respectively. The Phusion high fidelity DNA polymerase (Invitrogen) was used. The PCR product was digested with *XmaI* and *Not*I, purified, and cloned into the p123 vector digested with the same restriction enzymes. To generate SG200P*otefhos2* and SG200Δ*hos2*P*otefhos2* strains, p123-*hos2* was linearized with *Ssp*I and integrated into the SG200 or SG200Δ*hos2 ip* locus by homologous recombination.

For HA3 tagging of endogenous Hos2, the plasmid pUMa792Hos2 was generated. pUMa792Hos2 is a pUMa792 derivative (P. Müller and R. Kahmann, http://www.mikrobiologie.hhu.de/ustilago-community.html) in which a 1 Kb PCR-generated DNA fragment corresponding to the C-terminal part of the Hos2 open reading frame, has been cloned in frame with the three HA epitope repeats present in the plasmid. Additionally, a 1kb PCR-generated DNA fragment of the 3’UTR of Hos2 has also been cloned into the same plasmid, downstream of the hygromycin resistance cassette. To clone these fragments, the Gibson Assembly Cloning Kit (New England Biolabs) was used. The DHO915-DHO916 primer pair was used to amplify the 1 Kb DNA fragment of the C-terminal part of the Hos2 open reading frame. The DHO917-DHO918 primer pair was used to amplify the 1 Kb DNA fragment corresponding to the Hos2 3’UTR. Oligonucleotide design and enzymatic reactions were performed according to the manufacturer’s instructions. We used 0.1 pmol of each of the above mentioned DNA fragments together with 0.1 pmol of the DNA fragments resulting from the digestion of pUMA792 with *SfiI*. To generate the FB1Hos2HA3 strain, the corresponding 4694 bp DNA fragment, containing the HA3 tagged Hos2 C-terminal fragment, the hygromycin resistance cassette and the 1 Kb DNA fragment corresponding to the 3’UTR of Hos2, were amplified from pUMa792Hos2 with the oligonucleotides DHO919-DHO920 and transformed into *U*. *maydis* FB1 protoplasts. The high-fidelity Phusion DNA polymerase was used. Sequences of all these primers can be found in S2 Table. Successful cloning was verified by PCR, sequencing and western blot.

For RNA extractions of AB31 and FB1*Pcrg1fuz7DD*, cells were grown until OD600 = 0.5–0.8 and the *nar* and *crg* promoters induced as described above. 25 ml of AB31 or FB1*Pcrg1fuz7DD* induced cells (5 hours and 30 minutes induction) were pelleted in 50 ml tubes, washed once with sterile distilled water and frozen in liquid nitrogen. Samples were stored at-80°C. Cells were thawed on ice for 10 minutes and suspended in 2 ml TRIzol Reagent (Invitrogen). Cells were lysed by the addition of a 0.5 ml volume of acid-washed glass beads, vortexed 10 times for 20 seconds each and incubated at room temperature for 5 minutes. Each sample was divided into two 1.5 ml tubes, each containing 1 ml of the sample. 200 μl of chloroform was added to each tube, which were then inverted during 15 seconds and incubated at room temperature for 3 minutes. Samples were centrifuged at 11500 rpm for 15 minutes in a bench-top centrifuge at 4°C. 500 μl of the supernatant was transferred to new RNase-free 1.5 ml tubes. 500 μl of 2-propanol was added followed by incubation at room temperature for 10 minutes. Samples were centrifuged at 11500 rpm for 10 minutes. The 2-propanol was removed and 100 μl of 70% ethanol added. After 5 minutes of centrifugation at 11500 rpm and 4°C, the ethanol was removed and samples dried at 37°C for 10 minutes. Finally, samples were suspended in 50 μl of RNase-free distilled water and RNA concentration quantified using a Nanodrop spectrophotometer (ThermoFisher Scientific).

For RNA extractions of strains grown on charcoal plates, biomass was recovered, transferred to liquid nitrogen pre-chilled mortars and crushed to a powder. The processed material was then transferred into 50 ml tubes containing 3 ml of TRIzol Reagent (Invitrogen) and a 1 ml volume of acid-washed glass beads. RNA extraction was performed as described above.

### RT-qPCR

10 μg of each RNA sample was treated with rDNase I according to the manufacturer’s protocol in a final volume of 30 μl. Samples were incubated for 30 minutes at 37°C. 3 μl of ribonuclease inactivation reagent was then added to each sample and incubated for 2 minutes at room temperature with constant shaking. Samples were then centrifuged at 13000 rpm for 2 minutes. 15 μL of the supernatant were taken for further steps. 3 μl of each DNA-free RNA sample were used for retrotranscription by first mixing them with 1 μl of 10 mM dNTPs, 0.17 μl of random oligonucleotide and 8.83 μl of RNase-free water. Next, samples were heated to 65°C for 5 minutes and then cooled to 4°C for 1 minute in a thermocycler. Immediately, 4 μl of 5X first strand cDNA buffer, 1 μl of 0.1 M DTT, 1 μl of RNase inhibitor (Rnasin) and 1 μl of Superscript III reverse transcriptase (Invitrogen) were added to each sample. Samples were then heated to 65°C 1 h to allow retrotranscription to take place and finally held at 12°C. After retrotranscription, 90 μl of RNase-free water was added to each sample, to reach a final volume of 110 μl. For qPCR analysis, samples were diluted 200 times (4 μl of sample in 796 μl of milliQ water). Samples of non-retrotranscribed RNA were used as controls for genomic DNA contamination. For standard curve serial dilutions of wild-type cDNA in the corresponding inducing conditions were used. The constitutively expressed *ppi* gene was used as an internal control. The SYBR green method was used. *bE1*, *mfa1*, *pra1*, *prf1* and *ppi* oligonucleotide sequences for RT-qPCR have been described previously [69–71]. Samples were loaded in triplicate and three independent experiments were performed in all cases. For normalisation, the lowest expression value was set to 1. Logarithmic-transformed expression values were used to allow visualisation of pairwise comparisons that include highly divergent values, which were not clearly visible in raw data plots. For homogeneity logarithmic transformation was applied to every gene expression analysis.

### Western blot analyses

To extract proteins from *U*. *maydis*, 25 ml of exponentially growing *U*. *maydis* cells were washed once with water and frozen in liquid nitrogen. The frozen pellet was resuspended in 300 μl of Workman Extract Buffer (40mM HEPES-NaOH pH7.4, 350 mM NaCl, 0.1% NP40, 10% glycerol, 1mM PMSF, 1 μg/ml Pepstatin A, 1 μg/ml Bestatin, EDTA-free protease inhibitor tablets (Roche)) and transferred into 1.5 ml screw-cap tubes containing acid washed glass beads. Cells were fragmented using a FastPrep homogeniser (MP Biomedicals) with power set to 6.0 using 4 x 40” pulses at maximum speed with 3 minutes rest between each pulse. Dry-ice was used to cool down the machine at the beginning of the process. To recover the liquid fraction, the bottom of the 1.5 ml tube was drilled with a needle, placed into a 5 ml tube and centrifuged at 1000 rpm for 1 minute. Samples (supernatant and any pellet formed) were then transferred to a new 1.5 ml tube and centrifuged for 10 minutes at 13000 rpm and 4°C. The supernatant, containing total protein extract, was finally transferred into a new 1.5 ml tube. For extraction of total protein with the aim of detecting histone modifications, a workman extract buffer with a higher salt concentration (600 mM NaCl) was used. Protein concentration was measured using the Bradford protein assay. 60 μg of total protein was used for classical western blots. For western blots used to detect histone modifications, 20 μg of total protein was loaded. To detect histone acetylation, an H4K16Ac (Cell Signalling; E2B8W; Rabbit; 1:1000 in TBST 5% BSA) specific antibody was used. Total histone H3 was detected using an Anti-Histone H3 antibody (Merk-Millipore; clone AS3; 1:2500 in TBST 5% fat-free milk). α-tubulin was detected using a Monoclonal Anti-α-Tubulin antibody (Sigma; clone B-5–1–2; Mouse; 1:10000 in TBST 5% fat-free milk). For the histone assays, proteins were transferred from 15% acrylamide gels to PVDF membranes for 45 minutes at 90 V. Other western blots were carried out using 10% acrylamide gels, with proteins transferred to nitrocellulose membranes for 90 minutes at 90 V.

### Chromatin immunoprecipitation (ChIP)

50 ml of exponentially growing *U*. *maydis* cells (OD600 = 0.5–0.8) were cross-linked by adding 1% formaldehyde and incubated for 30 minutes with constant shaking at room temperature. Glycine was added to a final concentration of 250 mM and cultures incubated at room temperature for 10 minutes. Samples were then centrifuged for 4 minutes at 4500 rpm and 4°C and resuspended in 10 ml of ice-cold PBS 1X. Centrifugation and resuspension in PBS 1X was repeated once. Samples were then centrifuged for 4 minutes at 4500 rpm and 4°C, resuspended in 800 μl of PBS 1X and transferred to 2 ml screw-cap tubes. Samples were centrifuged at 6000 rpm for 2 minutes at room temperature, and the pellet frozen in liquid nitrogen and stored at-80°C. Pellets were thawed on ice for 5 minutes and resuspended in 800 μl of ice-cold LB140 (50 mM HEPES-KOH pH7.4, 140 mM NaCl, 1 mM EDTA, 1% Triton X-100, 0.1% sodium deoxycholate, supplemented with 1 mM PMSF, 1 μg/ml of pepstatin A, 1 μg/ml of bestatin, and 1X EDTA-free protease inhibitor tablets (Roche)). Acid-washed glass beads were added up to the meniscus of the liquid and cells were fragmented using the FastPrep homogeniser as described above but with 6 x 40” pulses at maximum speed with 3 minutes rest between each pulse. Dry-ice was used to cool down the machine both at the beginning and during the process). Samples were recovered by drilling a hole into the 2 ml screw-cap tube, which was introduced into a 5 ml tube, and centrifuged for 1 minute at 1000 rpm. Liquid and any pellet formed were transferred from the 5 ml tube into a new 1.5 ml tube. Samples were then centrifuged at maximum speed for 5 minutes at 4°C. Pellets, containing the chromatin insoluble fraction, were resuspended in 800 μl of LB140, and centrifuged at maximum speed for 5 minutes at 4°C. Supernatant was discarded, the pellet resuspended in 1.2 ml of LB140 and PMSF re-added. Samples were then sonicated in a Brandson sonicator, using an amplitude of 20% with 12 x 10” pulses separated by 50” rest periods. Under these conditions chromatin fragments had an average size of about 500 bp. 5 μl of the sonicated chromatin extract was used for determining protein concentration using the Bradford protein assay. 2% of the chromatin extract was kept as input, and frozen at-20°C. A total of 1 mg of chromatin extract was used for IP in a final volume of 500 μl. Volume was adjusted using LB140. For IP, to each 500 μl sample, 3 μg of anti-HA anti-body (abcam, ab9110) were used. Samples were incubated at 4°C for 16 hours on a rotating platform. Protein G sepharose beads (GE Healthcare) were pre-washed twice with distilled sterile water, twice with LB140 and resuspended in LB140. 50 μl of pre-washed beads were added to each sample, which were then incubated for 5 h at 4°C on a rotating platform. Samples were then centrifuged at 5000 rpm for 1 minute at room temperature. Supernatant was removed by aspiration and beads washed twice with 800 μl of each of the following buffers: (i) WB140 (50 mM HEPES-KOH pH7.4, 140 mM NaCl, 1 mM EDTA, 1% Triton X-100, 0.1% sodium deoxycholate); (ii) WB500 (50 mM HEPES-KOH pH7.4, 500 mM NaCl, 1 mM EDTA, 1% Triton X-100, 0.1% sodium deoxycholate); (iii) WBLiCl (10 mM Tris-HCl pH 7.4, 250 mM LiCl, 1 mM EDTA, 0.5% NP40, 0.5% sodium deoxycholate). The first wash was performed by adding 800 μl of the buffer, mixing by inverting the tube and centrifuging the cells at 5000 rpm for 1 minute at room temperature using a bench-top centrifuge. For the second wash, samples were incubated in the corresponding buffer for 5 minutes at room temperature with constant shaking, prior to centrifugation. Finally, cells were washed with TE10:1 (10mM Tris-HCl pH 7.4, 1mM EDTA) with a 5 minute incubation at room temperature. After centrifugation for 1 minute at 5000 rpm, supernatant was removed, the centrifugation repeated and the remaining TE10:1 supernatant completely removed. All buffers were ice cold. For elution, 105 μl of TES (50 mM Tris-HCl pH 7.4, 10 mM EDTA, 1%SDS) was added to each sample, incubated for 30 minutes at 65°C with occasional mixing. Samples were centrifuged at maximum speed for 1 minute at room temperature and 100 μl of supernatant were transferred into a new 1.5 ml tube. Another 105 μl of TES were added to the beads, incubated for 15 minutes at 65°C with occasional mixing. After centrifuging at maximum speed for 1 minute at room temperature, another 100 μl of the supernatant was transferred to the corresponding previous 1.5 ml tube, resulting in a final volume of 200 μl for each sample. To reverse the crosslinking, input samples were thawed at room temperature, the volume was adjusted to 50 μl with LB140, and 150 μl of TES added to each sample. Next, both input and IP chromatin extracts were incubated at 65°C for 16 hours. 250 μl of TE10:1, 1 μl of glycogen (20 mg/ml) and 7 μl of Proteinase K (20 mg/ml) were added to each sample and incubated for 2 h at 37°C. To extract and precipitate the DNA, 450 μl of phenol:chloroform:isoamyl alcohol (25:24:1) was added to each sample, followed by vortexing for 20 seconds and centrifugation at full speed for 5 minutes at room temperature. Supernatant was transferred to a new 1.5 ml tube. 450 μl of chloroform:isoamyl alcohol (24:1) was added to each sample, vortexed for 5 seconds and centrifuged at full speed for 2 minutes at room temperature. Supernatant was transferred into a new 1.5 ml tube. 12.5 μl of 5 M NaCl and 1 ml of ice-cold 100% ethanol were then added. Samples were incubated at-80°C for 2 h. A maximum of 4 samples were centrifuged at a time for 10 minutes at full speed and 4°C. Supernatant was removed and pellet washed with 1 ml of 70% ethanol. After removing the ethanol, pellets were dried at room temperature for 15 minutes. Finally, 100 μl of TE10:1 was added to each sample and incubated for 10 minutes at room temperature. For qPCR a 3 fold dilution of each immunoprecipitated sample and a 200-fold dilution of input (2%) samples were used. The oligonucleotides used for each amplicon were: (i) DHO1024 and DHO1025 for *prf1*–3.5 kb; (ii) DHO981 and DHO982 for *prf1*–2kb; (iii) DHO1022 and DHO1023 for *prf1*–1.5 kb (UAS); (iv) DHO983 and DHO984 for *prf1*–0.5 kb; (v) DHO985 and DHO986 for *prf1* ORF (5’); (vi) DHO991 and DHO992 for *bE1*; and (vii) DHO1001 and DHO1002 for *um01779*. Oligonucleotide sequences can be found in S2 Table. The oligonucleotide sequences for *mfa1*, *pra1*, *prf1* ORF (3’) and *ppi* amplicons have previously been described in [69, 70].

### Sequence alignment and domain structure

*U*. *maydis* Hos1, Hos2, Hos3, Hda1, Hda2 and Clr3 sequences were obtained from the MIPS *U*. *maydis* database (http://mips.gsf.de/genre/proj/ustilago/). *S*. *cerevisiae*, *S*. *pombe* and *C*. *albicans* HDAC sequences were obtained from SGD (http://www.yeastgenome.org/), PomBase (http://www.pombase.org/) and CGD (http://www.candidagenome.org) databases, respectively. Multiple sequence alignments and phylogenetic analyses were performed using MEGA5 [72]. Domain structure analysis was performed using the InterProScan Sequence Search tool from the European Bioinformatics Institute (http://www.ebi.ac.uk/). Pfam retrieved domains were used. Schematic representation of the retrieved domains was performed maintaining the proportions of each domain with respect to the whole protein sequence length.

### Microscopy

Images showing cell morphology, conjugation tube formation or *b*-dependent filamentation were taken using bright field Zeiss Apotome microscopes from the Centro Andaluz de Biología del Desarrollo (CABD) and from the Montpellier RIO Imaging (MRI) platform at the Centre de Recherche de Biochimie Macromoléculaire (CRBM). 40X and 63X objectives were used. Measurements of cell length and width were performed using ImageJ. Image acquisition, analysis and processing was carried out using Metamorph and Adobe Photoshop CS2.

### Statistical analysis

Data are expressed as means ±SD of at least triplicate samples. Statistical analysis and significance was assessed using GraphPad Prism 5 and considered significant if p-values were <0.05. t-test was used when comparing two means for differences. Fisher’s exact test was used to compare two or more groups with a categorical variable as the outcome. For multiple comparisons, one-way or two way ANOVA followed by Duncan’s new multiple range test was used. We performed Mann-Whitney tests (also known as Wilcoxon rank-sum test) to compare the distributions of disease symptoms induced by *U*. *maydis* strains.

### Accession numbers

*U*. *maydis* sequence data can be found in the NCBI protein libraries under accession numbers, XP_760381.1 for Hos1, XP_756808.1 for Hos2, XP_761874.1 for Hos3, XP_758212.1 for Hda1, XP_757499.1 for Hda2 and XP_758249.1 for Clr3. Other sequences used in this study have the following accession numbers: *S*. *cerevisiae* Hos1, NP_015393.1; Hos2, NP_011321.1; Hos3, NP_015209.1; Hda1, NP_014377.1; Rpd3, NP_014069.1; *S*. *pombe* Hos2, NP_594079.1; Clr3, NP_595104.1; Clr6, NP_595333.1; *C*. *albicans* Hos1, XP_723599.1; Hos2, XP_717660.1; Hos3, XP_720292.1; Hda1, XP_718271.1; Rpd3, XP_715765.1.

## Supporting Information

S1 Fig Schematic representation of the genetic control of the dimorphic switch and virulence in *U*. *maydis*. Pheromone-receptor recognition, as well as other environmental signals, promote the activation of cAMP-PKA and MAPK pathways. Transcriptional and posttranslational activation of the central Prf1 regulator is induced by these two pathways. Prf1 is an HMG transcription factor directly responsible for the activation of *a* and *b* mating-type genes. *a* genes encode the pheromone and pheromone-receptor proteins. Prf1-induced expression of *a* genes constitutes a positive feedback loop for mating between compatible partners, by promoting the formation of conjugation tubes via the MAP kinase cascade. *b* genes encode two transcription factors, bE and bW, that form a compatible heterodimer when expressed from different alleles, i.e., from different mating partners. The active bE/bW heterodimer promotes infective filament formation and virulence. Dashed coloured arrows represent external input signals that activate the cAMP-PKA and MAPK pathways. Dashed black arrows denote indirect transcriptional regulation. Continuous coloured arrows indicate input factors from the opposite mating-type cell. Continuous black arrows represent direct transcriptional control. The pointed black arrow indicates a positive feedback loop. Specific posttranslational modifications on Prf1, consisting of phosphorylations, are denoted by circled P symbols, coloured blue for cAMP phosphorylation and red for MAPK.
(TIF)

S2 Fig HDAC domains. Domain structure of class I and II HDACs in *Saccharomyces cerevisiae* (Sc), *Schizosaccharomyces pombe* (Sp), *Candida albicans* (Ca) and *Ustilago maydis* (Um). The HDAC domain is shown in red and the Arb2 domain specific of ScHda1-like histone deacetylases is shown in green.
(TIF)

S3 Fig Hos2 is a *bona fide* HDAC. (A) Western blot analysis of whole cell extracts of wild-type FB1 and FB1Δ*hos2* mutants grown to exponential phase in PD liquid medium. Blots were probed with antibodies against acetylated H4K16, total H3 as a control for total nucleosome content and α-tubulin as a loading control. Numbers indicate molecular weight in kDa. (B) Serial dilution assay of single HDAC mutants grown for 2 days at 28°C on control YPD plates or on YPD plates supplemented with 0.25 μg/ml of TSA.
(TIF)

S4 Fig Morphology and growth of Δ*hos2* cells. (A) Doubling time of the indicated strains during exponential growth in YESPL. Mean values and SDs from three independent experiments are shown. Not statistically significant (ns) differences were found (t-test, *p*>0.05). (B) Cell length of the indicated strains. Each point corresponds to the measurement of a single cell. Three independent experiments, each comprising of 100 cell measurements were performed. The red line indicates the mean value of the three independent biological experiments. Not statistically significant (ns) differences were found (Duncan’s new multiple range test, *p*>0.05). (C) Cell width measurements of the indicated strains were performed as described above for cell length. Not statistically significant (ns) differences were found (Duncan’s new multiple range test, *p*>0.05). (D) Optical microscopy images of FB2 and FB2Δ*hos2* cells during exponential growth in YEPSL liquid medium. (E) Quantification of the number of buds per cell in wild-type and Δ*hos2* mutants in the FB2 background. Mean values and SDs from three independent experiments are shown. Statistically significant differences are indicated (Fisher’s exact test, ** is used for *p*<0.01; **** for *p*<0.0001).
(TIF)

S5 Fig Mating phenotypes of *hos1*, *hos3*, *hda2* and *clr3* single mutants. Indicated strains were grown in YEPSL medium to exponential phase and then spotted alone or in combination with other strains on PD charcoal plates and incubated for 24 hours at 25°C.
(TIF)

S6 Fig Effect of TSA on conjugation tube formation. (A) Quantification of the effect of TSA on conjugation tube formation after a2 pheromone addition. FB1 cells were grown in CMD until exponential phase and treated either with 0.5 μg/ml of TSA for 2 hours, or with DMSO. a2 pheromone was then added to each culture for 5 hours before counting conjugation tube formation under the microscope. Mean values and SDs from three independent experiments are shown. Total number of cells counted is indicated above each column. *** denotes a statistically significant difference with *p*< 0.001 (t-test). (B) FB1 cells were grown in CMD until exponential phase and treated either with TSA to a final concentration of 0.5 μg/ml or DMSO for 7 hours. These cultures were assayed for cell viability, by plating 200 cells onto YPD plates and counting the number of colonies after 2 days incubation at 28°C. Mean values and SDs from three independent experiments are shown. Not statistically significant (ns) differences were found in any pairwise comparison (Duncan’s new multiple range test, *p*>0.05).
(TIF)

S7 Fig Overexpression of *hos2*. (A) Expression of *hos2* relative to *ppi* in the indicated strains. Mean values and SDs from three independent experiments, each containing three technical replicates, are shown. Values are normalised to one of the biological replicates of the sample with the lowest expression value (SG200) that is assigned a value of 1. **** denotes a statistically significant difference with *p*<0.0001 (t-test) (B) Filamentation of the indicated solopathogenic strains grown on PD charcoal plates for 48 h at 25°C. (C) Quantification of the conjugation tube formation capacity of the indicated strains after 5 h exposed to a2 pheromone. Mean values and SDs from three independent experiments are shown. The total number of cells counted is indicated above each column. * denotes a statistically significant difference with *p*<0.05 (Duncan’s new multiple range test). (D) Quantification of plant symptoms infected with the indicated strains 14 days post-infection (dpi). Mean values of three independent experiments are shown. The total number of infected plants is indicated above each column. * denotes a statistically significant difference with *p*<0.05 (Mann-Whitney test)
(TIF)

S8 Fig The Hos2-HA3 allele is functional. Exponentially growing cultures of the FB1 and FB1Hos2-HA3 strains were treated with a2 pheromone for 5 hours and conjugation tube formation quantified. Mean values and SDs from three independent experiments are shown. Total numbers of counted cells are indicated above each column. ns denotes not statistically significant difference (*p*>0.05, t-test).
(TIF)

S9 Fig Expression of Hos2 under different growth conditions. (A) *hos2* expression level in the SG200 wild-type strain grown in CM or PD liquid media to early (E) or late (L) exponential phase. ns denotes not statistically significant difference (Duncan’s new multiple range test, *p*<0.05). (B) *hos2* expression levels in the SG200 strain grown in CM liquid medium with 1% glucose as carbon source, or on CM charcoal plates. (C) *hos2* expression upon activation of the MAPK pathway. Induction of the MAPKK Fuz7 was performed as described in the Methods section. (D) *hos2* expression level upon induction of a compatible bE1/bW2 heterodimer in the AB31 background (see Methods). (E) Effect of cAMP addition on *hos2* expression. 6 mM of cAMP was added to exponentially growing cultures of FB1 wild-type strain in PD broth. RNA extraction was performed 8 hours after cAMP addition. In A-E *hos2* expression was quantified by RT-qPCR. Mean values and SDs from three independent experiments, each consisting of three technical replicates, are shown. In B-E ns denotes not statistically significant differences (t-test, *p*>0.05) (F) Hos2-HA3 protein levels from chromatin extracts used for ChIP, with or without the addition of cAMP. Tubulin was used as a loading control.
(TIF)

S10 Fig ChIP analysis of the *prf1* locus. (A) Overview of the promoter and open reading frame of the *prf1* gene. The probes used for qPCR analysis of the Hos2-HA3 ChIP experiment are indicated. Arrows represent the primers used for each specific amplicon. Numbers above the arrows indicate the specific coordinates relative to the first *prf1* ORF ATG, with the adenine considered position +1. The name of each amplicon is indicated underneath and is used in subsequent panels of this figure. (B) ChIP analysis using an anti-HA antibody on chromatin extracts from either an untagged (blue) or a Hos2-HA3 (red) strain, grown in PD broth medium. Inmunoprecipitated DNA was analysed by qPCR, amplifying the regions indicated on the *x* axis and in panel A. Values correspond to the amount of DNA recovered by the HA IP divided by the amount of DNA in the corresponding input extract. Mean values and SDs from four independent experiments, each with three technical replicates, are shown. * denotes statistically significant differences (Duncan’s new multiple range test, *p*<0.05). (C) ChIP analysis was performed and analysed as in (A), except strains were grown in PD with or without the addition of 6 mM cAMP for 8 hours. For simplicity, values for the untagged strains are not shown, but were identical to those shown in (A) and did not vary upon cAMP addition. Statistically significant (*) and not significant (ns) differences are shown (Duncan’s new multiple range test, *p*<0.05).
(TIF)

S1 Table *U*. *maydis* strains used in this study. (DOC)

S2 Table Primers used in this study. (DOC)

## References

[1] Schulz B, Banuett F, Dahl M, Schlesinger R, Schafer W, et al (1990) The b alleles of U. maydis, whose combinations program pathogenic development, code for polypeptides containing a homeodomain-related motif. Cell 60(2): 295–306. 1967554 10.1016/0092-8674(90)90744-y

[2] Lo HJ, Kohler JR, DiDomenico B, Loebenberg D, Cacciapuoti A, et al (1997) Nonfilamentous C. albicans mutants are avirulent. Cell 90(5): 939–949. 9298905 10.1016/s0092-8674(00)80358-x

[3] Lin X, Huang JC, Mitchell TG, Heitman J. (2006) Virulence attributes and hyphal growth of C. neoformans are quantitative traits and the MATalpha allele enhances filamentation. PLoS Genet 2(11): e187 10.1371/journal.pgen.0020187 17112316 PMC1636697

[4] Nadal M, Garcia-Pedrajas MD, Gold SE. (2008) Dimorphism in fungal plant pathogens. FEMS Microbiol Lett 284(2): 127–134. 10.1111/j.1574–6968.2008.01173.x 18479435

[5] Braun BR, Johnson AD. (2000) TUP1, CPH1 and EFG1 make independent contributions to filamentation in candida albicans. Genetics 155(1): 57–67. 10790384 10.1093/genetics/155.1.57 PMC1461068

[6] Sanchez-Martinez C, Perez-Martin J. (2001) Dimorphism in fungal pathogens: Candida albicans and ustilago maydis—similar inputs, different outputs. Curr Opin Microbiol 4(2): 214–221. 11282479 10.1016/s1369-5274(00)00191-0

[7] Liu H. (2002) Co-regulation of pathogenesis with dimorphism and phenotypic switching in candida albicans, a commensal and a pathogen. Int J Med Microbiol 292(5–6): 299–311. 12452278 10.1078/1438-4221-00215

[8] Brefort T, Doehlemann G, Mendoza-Mendoza A, Reissmann S, Djamei A, et al (2009) Ustilago maydis as a pathogen. Annu Rev Phytopathol 47: 423–445. 10.1146/annurev-phyto-080508–081923 19400641

[9] Bolker M. (2001) Ustilago maydis—a valuable model system for the study of fungal dimorphism and virulence. Microbiology 147(Pt 6): 1395–1401. 11390671 10.1099/00221287-147-6-1395

[10] Snetselaar KM, Mims CW. (1992) Sporidial fusion and infection of maize seedlings by the smut fungus ustilago maydis. Mycologia 84(2): 193–203.

[11] Snetselaar KM, Mims CW. (1994) Light and electron microscopy of ustilago maydis hyphae in maize. Mycol Res 98(3): 347–355.

[12] Mendoza-Mendoza A, Berndt P, Djamei A, Weise C, Linne U, et al (2009) Physical-chemical plant-derived signals induce differentiation in ustilago maydis. Mol Microbiol 71(4): 895–911. 10.1111/j.1365–2958.2008.06567.x 19170880

[13] Banuett F, Herskowitz I. (1996) Discrete developmental stages during teliospore formation in the corn smut fungus, ustilago maydis. Development 122(10): 2965–2976. 8898211 10.1242/dev.122.10.2965

[14] Bolker M, Urban M, Kahmann R. (1992) The a mating type locus of U. maydis specifies cell signaling components. Cell 68(3): 441–450. 1310895 10.1016/0092-8674(92)90182-c

[15] Spellig T, Bolker M, Lottspeich F, Frank RW, Kahmann R. (1994) Pheromones trigger filamentous growth in ustilago maydis. EMBO J 13(7): 1620–1627. 8157001 10.1002/j.1460-2075.1994.tb06425.x PMC394992

[16] Gillissen B, Bergemann J, Sandmann C, Schroeer B, Bolker M, et al (1992) A two-component regulatory system for self/non-self recognition in ustilago maydis. Cell 68(4): 647–657. 1739973 10.1016/0092-8674(92)90141-x

[17] Kamper J, Reichmann M, Romeis T, Bolker M, Kahmann R. (1995) Multiallelic recognition: Nonself-dependent dimerization of the bE and bW homeodomain proteins in ustilago maydis. Cell 81(1): 73–83. 7720075 10.1016/0092-8674(95)90372-0

[18] Hartmann HA, Kahmann R, Bolker M. (1996) The pheromone response factor coordinates filamentous growth and pathogenicity in ustilago maydis. EMBO J 15(7): 1632–1641. 8612587 PMC450074

[19] Hartmann HA, Kruger J, Lottspeich F, Kahmann R. (1999) Environmental signals controlling sexual development of the corn smut fungus ustilago maydis through the transcriptional regulator Prf1. Plant Cell 11(7): 1293–1306. 10402430 10.1105/tpc.11.7.1293 PMC144278

[20] Muller P, Aichinger C, Feldbrugge M, Kahmann R. (1999) The MAP kinase kpp2 regulates mating and pathogenic development in ustilago maydis. Mol Microbiol 34(5): 1007–1017. 10594825 10.1046/j.1365-2958.1999.01661.x

[21] Kaffarnik F, Muller P, Leibundgut M, Kahmann R, Feldbrugge M. (2003) PKA and MAPK phosphorylation of Prf1 allows promoter discrimination in ustilago maydis. EMBO J 22(21): 5817–5826. 10.1093/emboj/cdg554 14592979 PMC275411

[22] Muller P, Weinzierl G, Brachmann A, Feldbrugge M, Kahmann R. (2003) Mating and pathogenic development of the smut fungus ustilago maydis are regulated by one mitogen-activated protein kinase cascade. Eukaryot Cell 2(6): 1187–1199. 14665454 10.1128/EC.2.6.1187-1199.2003 PMC326639

[23] Kruger J, Loubradou G, Regenfelder E, Hartmann A, Kahmann R. (1998) Crosstalk between cAMP and pheromone signalling pathways in ustilago maydis. Mol Gen Genet 260(2–3): 193–198. 9862471 10.1007/s004380050885

[24] Brefort T, Muller P, Kahmann R. (2005) The high-mobility-group domain transcription factor Rop1 is a direct regulator of prf1 in ustilago maydis. Eukaryot Cell 4(2): 379–391. 10.1128/EC.4.2.379–391.2005 15701800 PMC549323

[25] Mendoza-Mendoza A, Eskova A, Weise C, Czajkowski R, Kahmann R. (2009) Hap2 regulates the pheromone response transcription factor prf1 in ustilago maydis. Mol Microbiol 72(3): 683–698. 10.1111/j.1365–2958.2009.06676.x 19400774

[26] Garrido E, Voss U, Muller P, Castillo-Lluva S, Kahmann R, et al (2004) The induction of sexual development and virulence in the smut fungus ustilago maydis depends on Crk1, a novel MAPK protein. Genes Dev 18(24): 3117–3130. 10.1101/gad.314904 15601825 PMC535921

[27] Elias-Villalobos A, Fernandez-Alvarez A, Ibeas JI. (2011) The general transcriptional repressor Tup1 is required for dimorphism and virulence in a fungal plant pathogen. PLoS Pathog 7(9): e1002235 10.1371/journal.ppat.1002235 21909277 PMC3164652

[28] Pijnappel WW, Schaft D, Roguev A, Shevchenko A, Tekotte H, et al (2001) The S. cerevisiae SET3 complex includes two histone deacetylases, Hos2 and Hst1, and is a meiotic-specific repressor of the sporulation gene program. Genes Dev 15(22): 2991–3004. 10.1101/gad.207401 11711434 PMC312828

[29] Wang A, Kurdistani SK, Grunstein M. (2002) Requirement of Hos2 histone deacetylase for gene activity in yeast. Science 298(5597): 1412–1414. 10.1126/science.1077790 12434058

[30] Hang M, Smith MM. (2011) Genetic analysis implicates the Set3/Hos2 histone deacetylase in the deposition and remodeling of nucleosomes containing H2A.Z. Genetics 187(4): 1053–1066. 10.1534/genetics.110.125419 21288874 PMC3070515

[31] Kim T, Buratowski S. (2009) Dimethylation of H3K4 by Set1 recruits the Set3 histone deacetylase complex to 5' transcribed regions. Cell 137(2): 259–272. 10.1016/j.cell.2009.02.045 19379692 PMC2802783

[32] Hnisz D, Majer O, Frohner IE, Komnenovic V, Kuchler K. (2010) The Set3/Hos2 histone deacetylase complex attenuates cAMP/PKA signaling to regulate morphogenesis and virulence of candida albicans. PLoS Pathog 6(5): e1000889 10.1371/journal.ppat.1000889 20485517 PMC2869326

[33] Ding SL, Liu W, Iliuk A, Ribot C, Vallet J, et al (2010) The tig1 histone deacetylase complex regulates infectious growth in the rice blast fungus magnaporthe oryzae. Plant Cell 22(7): 2495–2508. 10.1105/tpc.110.074302 20675574 PMC2929099

[34] Baidyaroy D, Brosch G, Ahn JH, Graessle S, Wegener S, et al (2001) A gene related to yeast HOS2 histone deacetylase affects extracellular depolymerase expression and virulence in a plant pathogenic fungus. Plant Cell 13(7): 1609–1624. 11449054 10.1105/TPC.010168 PMC139552

[35] Reichmann M, Jamnischek A, Weinzierl G, Ladendorf O, Huber S, et al (2002) The histone deacetylase Hda1 from ustilago maydis is essential for teliospore development. Mol Microbiol 46(4): 1169–1182. 12421320 10.1046/j.1365-2958.2002.03238.x

[36] Gonzalez-Prieto JM, Dominguez A, Rosas-Quijano R, Cervantes-Chavez JA, Ruiz-Herrera J. (2004) Isolation and molecular analysis of Umhda2 a gene encoding a histone deacetylase from ustilago maydis. DNA Seq 15(1): 44–50. 15354354 10.1080/10425170310001652192

[37] Wirén M, Silverstein RA, Sinha I, Walfridsson J, Lee H, et al (2005) Genomewide analysis of nucleosome density histone acetylation and HDAC function in fission yeast. The EMBO Journal 24(16): 2906–2918. 10.1038/sj.emboj.7600758 16079916 PMC1187943

[38] Olsson TG, Ekwall K, Allshire RC, Sunnerhagen P, Partridge JF, et al (1998) Genetic characterisation of hda1+, a putative fission yeast histone deacetylase gene. Nucleic Acids Res 26(13): 3247–3254. 9628926 10.1093/nar/26.13.3247 PMC147680

[39] Kim YB, Honda A, Yoshida M, Horinouchi S. (1998) Phd1+, a histone deacetylase gene of schizosaccharomyces pombe, is required for the meiotic cell cycle and resistance to trichostatin A. FEBS Letters 436(2): 193 9781677 10.1016/s0014-5793(98)01124-7

[40] Bolker M, Genin S, Lehmler C, Kahmann R. (1995) Genetic regulation of mating, and dimorphism in ustilago maydis. Can J Bot 73: 320–325.

[41] Brachmann A, Weinzierl G, Kamper J, Kahmann R. (2001) Identification of genes in the bW/bE regulatory cascade in ustilago maydis. Mol Microbiol 42(4): 1047–1063. 11737646 10.1046/j.1365-2958.2001.02699.x

[42] Flor-Parra I, Vranes M, Kamper J, Perez-Martin J. (2006) Biz1, a zinc finger protein required for plant invasion by ustilago maydis, regulates the levels of a mitotic cyclin. Plant Cell 18(9): 2369–2387. 10.1105/tpc.106.042754 16905655 PMC1560913

[43] Heimel K, Scherer M, Vranes M, Wahl R, Pothiratana C, et al (2010) The transcription factor Rbf1 is the master regulator for b-mating type controlled pathogenic development in ustilago maydis. PLoS Pathog 6(8): e1001035 10.1371/journal.ppat.1001035 20700446 PMC2916880

[44] Watson AD, Edmondson DG, Bone JR, Mukai Y, Yu Y, et al (2000) Ssn6-Tup1 interacts with class I histone deacetylases required for repression. Genes Dev 14(21): 2737–2744. 11069890 10.1101/gad.829100 PMC317033

[45] Davie JK, Edmondson DG, Coco CB, Dent SY. (2003) Tup1-Ssn6 interacts with multiple class I histone deacetylases in vivo. J Biol Chem 278(50): 50158–50162. 10.1074/jbc.M309753200 14525981

[46] Barrett KJ, Gold SE, Kronstad JW. (1993) Identification and complementation of a mutation to constitutive filamentous growth in ustilago maydis. Mol Plant Microbe Interact 6(3): 274–283. 8324246 10.1094/mpmi-6-274

[47] Gold S, Duncan G, Barrett K, Kronstad J. (1994) cAMP regulates morphogenesis in the fungal pathogen ustilago maydis. Genes Dev 8(23): 2805–2816. 7995519 10.1101/gad.8.23.2805

[48] Durrenberger F, Wong K, Kronstad JW. (1998) Identification of a cAMP-dependent protein kinase catalytic subunit required for virulence and morphogenesis in ustilago maydis. Proc Natl Acad Sci U S A 95(10): 5684–5689. 9576944 10.1073/pnas.95.10.5684 PMC20439

[49] Mayorga ME, Gold SE. (1998) Characterization and molecular genetic complementation of mutants affecting dimorphism in the fungus ustilago maydis. Fungal Genet Biol 24(3): 364–376. 10.1006/fgbi.1998.1078 9756717

[50] Hnisz D, Bardet AF, Nobile CJ, Petryshyn A, Glaser W, et al (2012) A histone deacetylase adjusts transcription kinetics at coding sequences during candida albicans morphogenesis. PLoS Genetics 8(12): e1003118 10.1371/journal.pgen.1003118 23236295 PMC3516536

[51] Robbins N, Leach MD, Cowen LE. (2012) Lysine deacetylases Hda1 and Rpd3 regulate Hsp90 function thereby governing fungal drug resistance. Cell Rep 2(4): 878–888. 10.1016/j.celrep.2012.08.035 23041319 PMC3607219

[52] Haberland M, Carrer M, Mokalled MH, Montgomery RL, Olson EN. (2010) Redundant control of adipogenesis by histone deacetylases 1 and 2. J Biol Chem 285(19): 14663–14670. 10.1074/jbc.M109.081679 20190228 PMC2863240

[53] Barrales RR, Korber P, Jimenez J, Ibeas JI. (2012) Chromatin modulation at the FLO11 promoter of saccharomyces cerevisiae by HDAC and swi/snf complexes. Genetics 191(3): 791–803. 10.1534/genetics.112.140301 22542969 PMC3389975

[54] Zacchi LF, Schulz WL, Davis DA. (2010) HOS2 and HDA1 encode histone deacetylases with opposing roles in candida albicans morphogenesis. PLoS One 5(8): e12171 10.1371/journal.pone.0012171 20730094 PMC2921335

[55] Izawa M, Takekawa O, Arie T, Teraoka T, Yoshida M, et al (2009) Inhibition of histone deacetylase causes reduction of appressorium formation in the rice blast fungus magnaporthe oryzae. J Gen Appl Microbiol 55(6): 489–498. 20118613 10.2323/jgam.55.489

[56] Li Y, Wang C, Liu W, Wang G, Kang Z, et al (2011) The HDF1 histone deacetylase gene is important for conidiation, sexual reproduction, and pathogenesis in fusarium graminearum. Mol Plant Microbe Interact 24(4): 487–496. 10.1094/MPMI-10–10–0233 21138346

[57] Wong KH, Struhl K. (2011) The Cyc8-Tup1 complex inhibits transcription primarily by masking the activation domain of the recruiting protein. Genes Dev 25(23): 2525–2539. 10.1101/gad.179275.111 22156212 PMC3243062

[58] Parnell EJ, Stillman DJ. (2011) Shields up: The Tup1-Cyc8 repressor complex blocks coactivator recruitment. Genes Dev 25(23): 2429–2435. 10.1101/gad.181768.111 22156205 PMC3243054

[59] Martinez-Salgado JL, Leon-Ramirez CG, Pacheco AB, Ruiz-Herrera J, de la Rosa A P. (2013) Analysis of the regulation of the ustilago maydis proteome by dimorphism, pH or MAPK and GCN5 genes. J Proteomics 79: 251–262. 10.1016/j.jprot.2012.12.022 23305952

[60] González-Prieto JM, Rosas-Quijano R, Domínguez A, Ruiz-Herrera J. (2014) The UmGcn5 gene encoding histone acetyltransferase from ustilago maydis is involved in dimorphism and virulence. Fungal Genetics and Biology: FG & B 71: 86–95. 10.1016/j.fgb.2014.09.002 25242418

[61] Robyr D, Suka Y, Xenarios I, Kurdistani SK, Wang A, et al (2002) Microarray deacetylation maps determine genome-wide functions for yeast histone deacetylases. Cell 109(4): 437–446. 12086601 10.1016/s0092-8674(02)00746-8

[62] van Werven FJ, Neuert G, Hendrick N, Lardenois A, Buratowski S, et al (2012) Transcription of two long noncoding RNAs mediates mating-type control of gametogenesis in budding yeast. Cell 150(6): 1170–1181. 10.1016/j.cell.2012.06.049 22959267 PMC3472370

[63] Lu Y, Su C, Wang A, Liu H. (2011) Hyphal development in candida albicans requires two temporally linked changes in promoter chromatin for initiation and maintenance. PLoS Biol 9(7): e1001105 10.1371/journal.pbio.1001105 21811397 PMC3139633

[64] Sambrook J, Frish E, Maniatis T. (1989) Molecular cloning: A laboratorymanual. New York: Cold Spring Harbour Laboratory Press.

[65] Holliday R. (1974) Ustilago maydis New York, USA: Plenum Press: Handbook of Genetics. 575 p.

[66] Kamper J. (2004) A PCR-based system for highly efficient generation of gene replacement mutants in ustilago maydis. Mol Genet Genomics 271(1): 103–110. 10.1007/s00438–003–0962–8 14673645

[67] Brachmann A, Konig J, Julius C, Feldbrugge M. (2004) A reverse genetic approach for generating gene replacement mutants in ustilago maydis. Mol Genet Genomics 272(2): 216–226. 10.1007/s00438–004–1047-z 15316769

[68] Wedlich-Soldner R, Bolker M, Kahmann R, Steinberg G. (2000) A putative endosomal t-SNARE links exo- and endocytosis in the phytopathogenic fungus ustilago maydis. EMBO J 19(9): 1974–1986. 10.1093/emboj/19.9.1974 10790364 PMC305698

[69] Scherer M, Heimel K, Starke V, Kamper J. (2006) The Clp1 protein is required for clamp formation and pathogenic development of ustilago maydis. Plant Cell 18(9): 2388–2401. 10.1105/tpc.106.043521 16920779 PMC1560919

[70] Zarnack K, Eichhorn H, Kahmann R, Feldbrugge M. (2008) Pheromone-regulated target genes respond differentially to MAPK phosphorylation of transcription factor Prf1. Mol Microbiol 69(4): 1041–1053. 10.1111/j.1365–2958.2008.06345.x 18627457

[71] Heimel K, Scherer M, Schuler D, Kamper J. (2010) The ustilago maydis Clp1 protein orchestrates pheromone and b-dependent signaling pathways to coordinate the cell cycle and pathogenic development. Plant Cell 22(8): 2908–2922. 10.1105/tpc.110.076265 20729384 PMC2947178

[72] Tamura K, Peterson D, Peterson N, Stecher G, Nei M, et al (2011) MEGA5: Molecular evolutionary genetics analysis using maximum likelihood, evolutionary distance, and maximum parsimony methods. Mol Biol Evol 28(10): 2731–2739. 10.1093/molbev/msr121 21546353 PMC3203626

