# Supplementary figures and images for: The Hos2 Histone Deacetylase Controls Ustilago maydis Virulence through Direct Regulation of Mating-Type Genes
Source: PLoS Pathog. 2015 Aug 28;11(8):e1005134. doi: 10.1371/journal.ppat.1005134 (PMC4552784; doi:10.1371/journal.ppat.1005134)

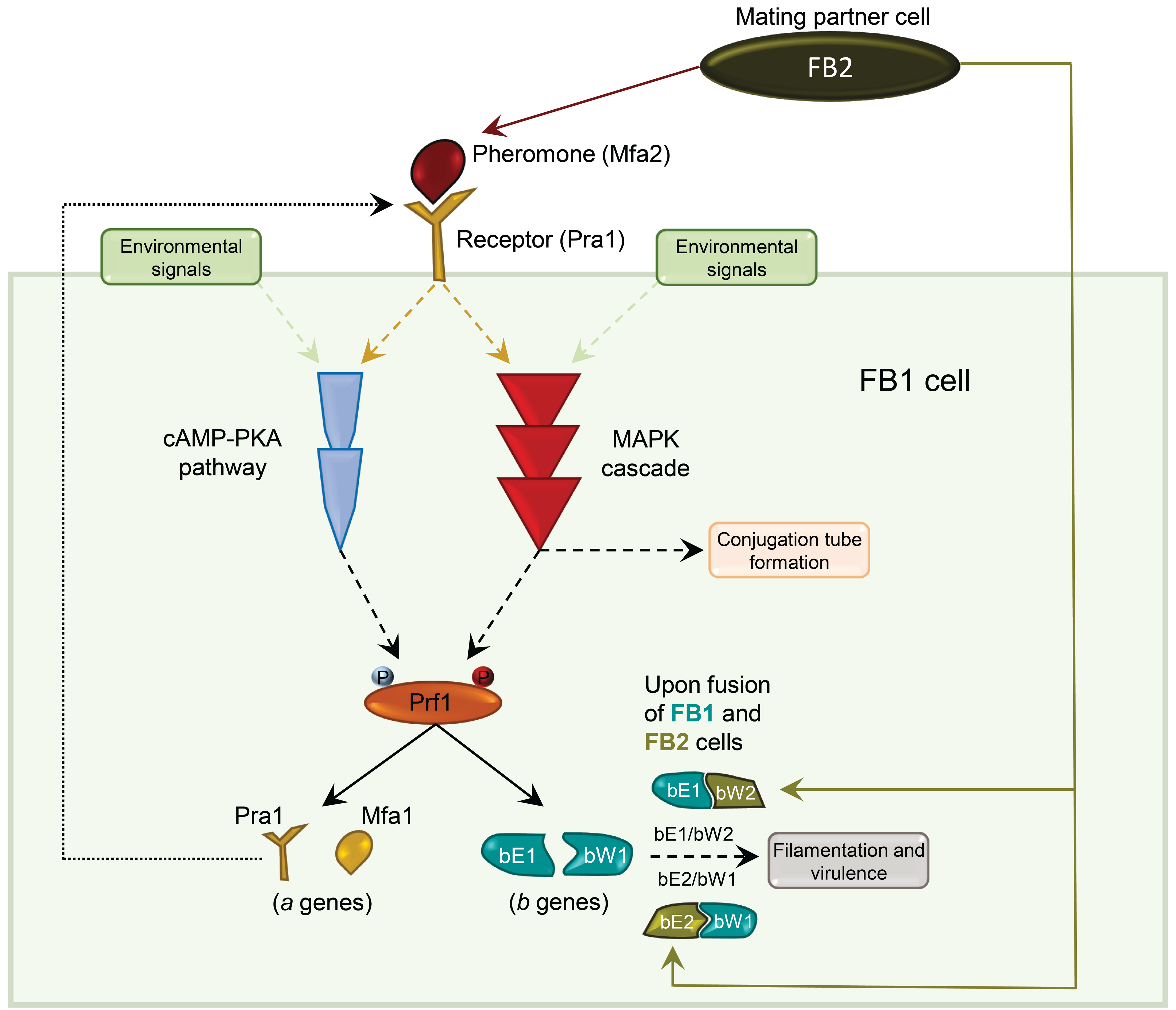

Supplement: S1 Fig — Pheromone-receptor recognition, as well as other environmental signals, promote the activation of cAMP-PKA and MAPK pathways. Transcriptional and posttranslational activation of the central Prf1 regulator is induced by these two pathways. Prf1 is an HMG transcription factor directly responsible for the activation of a and b mating-type genes. a genes encode the pheromone and pheromone-receptor proteins. Prf1-induced expression of a genes constitutes a positive feedback loop for mating between compatible partners, by promoting the formation of conjugation tubes via the MAP kinase cascade. b genes encode two transcription factors, bE and bW, that form a compatible heterodimer when expressed from different alleles, i.e., from different mating partners. The active bE/bW heterodimer promotes infective filament formation and virulence. Dashed coloured arrows represent external input signals that activate the cAMP-PKA and MAPK pathways. Dashed black arrows denote indirect transcriptional regulation. Continuous coloured arrows indicate input factors from the opposite mating-type cell. Continuous black arrows represent direct transcriptional control. The pointed black arrow indicates a positive feedback loop. Specific posttranslational modifications on Prf1, consisting of phosphorylations, are denoted by circled P symbols, coloured blue for cAMP phosphorylation and red for MAPK. (TIF) [file ppat.1005134.s001.tif]

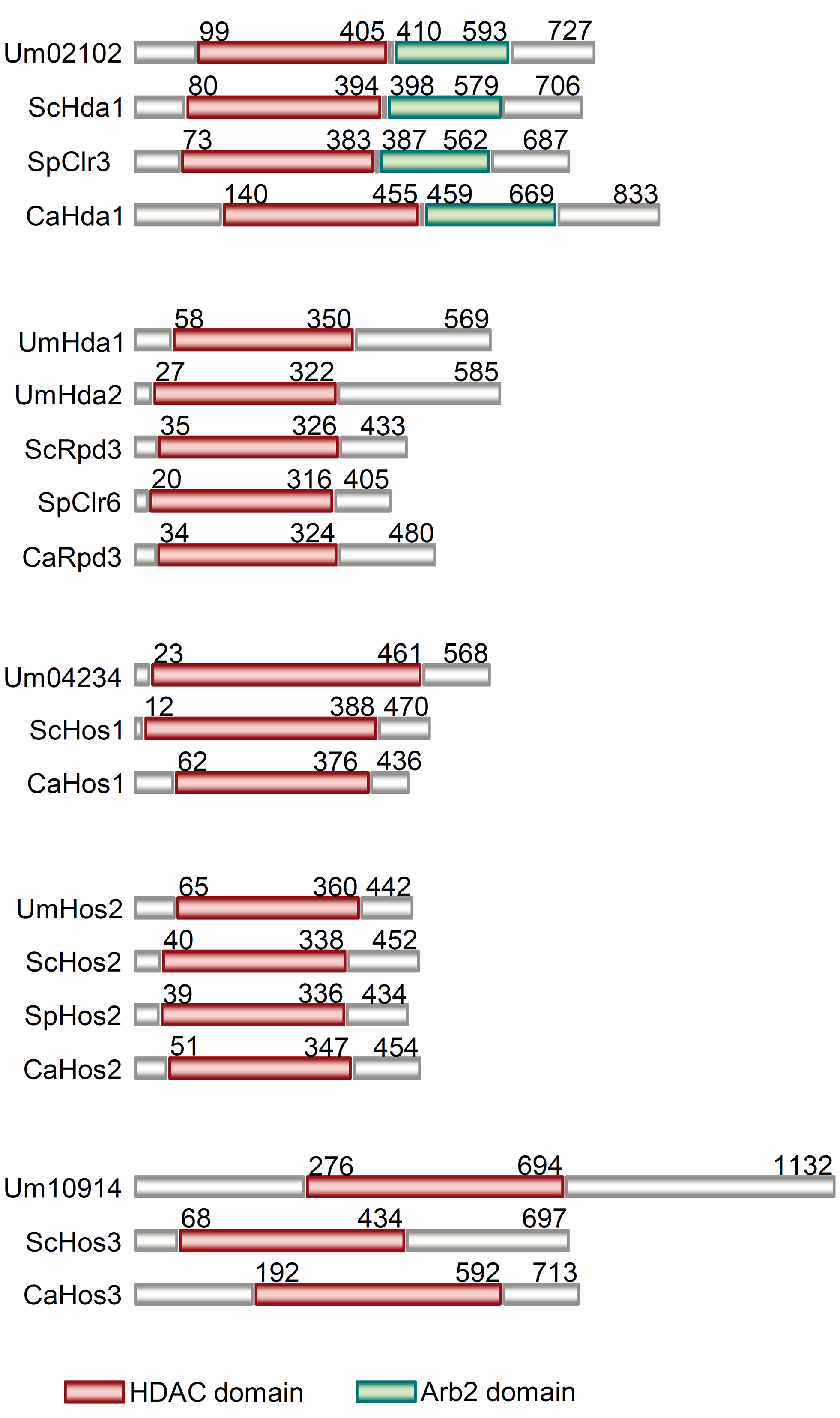

Supplement: S2 Fig — Domain structure of class I and II HDACs in Saccharomyces cerevisiae (Sc), Schizosaccharomyces pombe (Sp), Candida albicans (Ca) and Ustilago maydis (Um). The HDAC domain is shown in red and the Arb2 domain specific of ScHda1-like histone deacetylases is shown in green. (TIF) [file ppat.1005134.s002.tif]

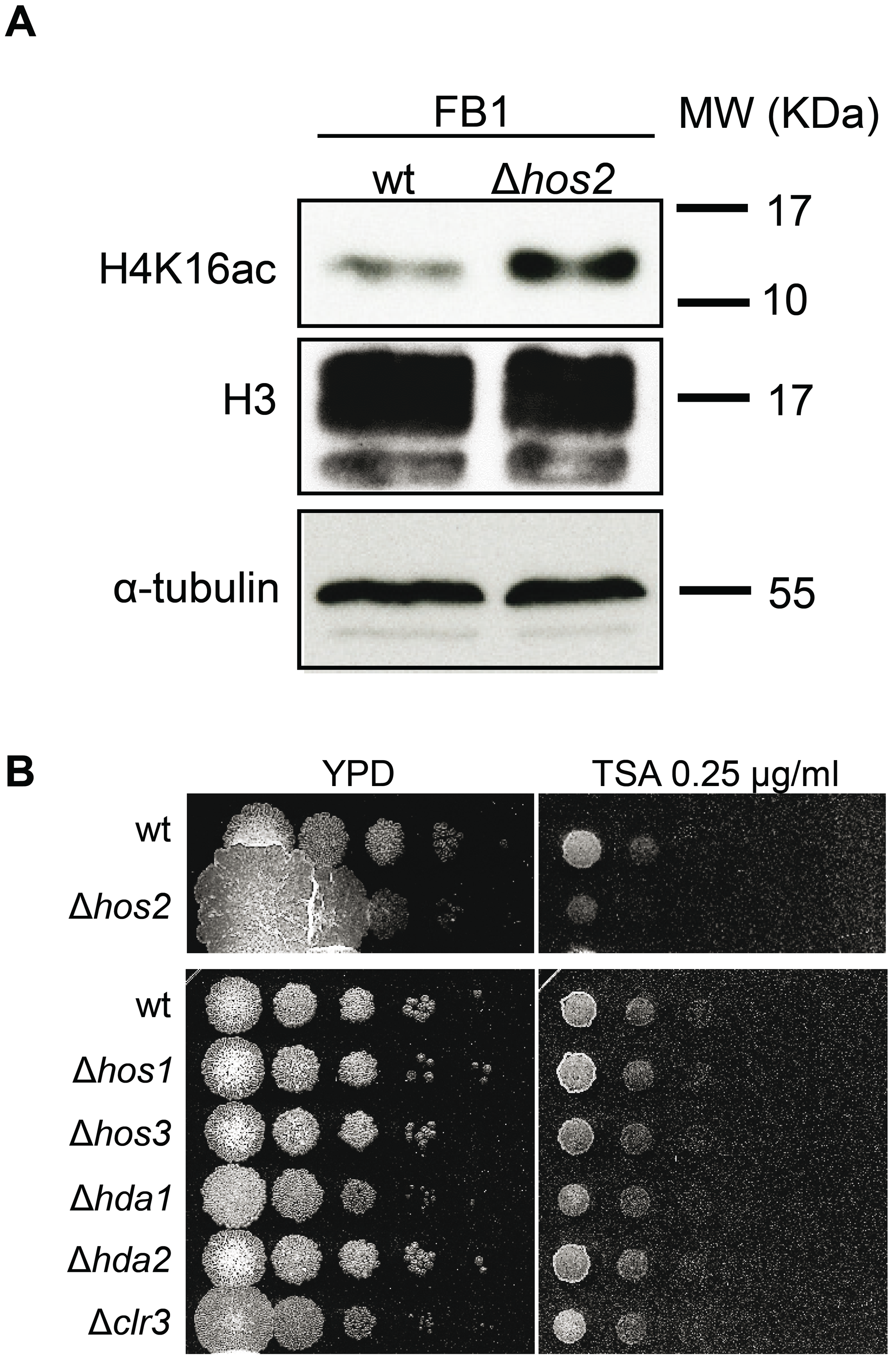

Supplement: S3 Fig — (A) Western blot analysis of whole cell extracts of wild-type FB1 and FB1Δhos2 mutants grown to exponential phase in PD liquid medium. Blots were probed with antibodies against acetylated H4K16, total H3 as a control for total nucleosome content and α-tubulin as a loading control. Numbers indicate molecular weight in kDa. (B) Serial dilution assay of single HDAC mutants grown for 2 days at 28°C on control YPD plates or on YPD plates supplemented with 0.25 μg/ml of TSA. (TIF) [file ppat.1005134.s003.tif]

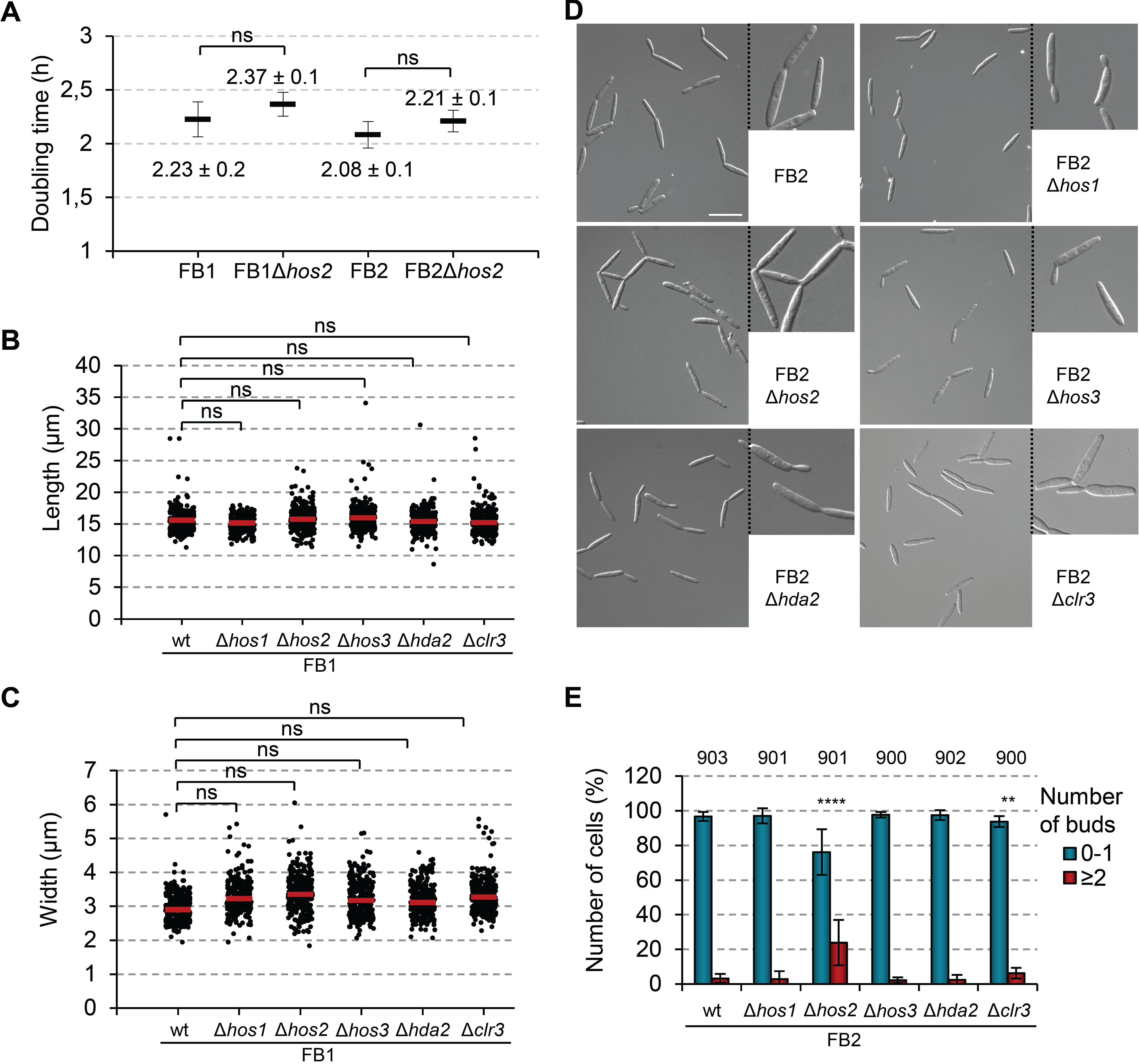

Supplement: S4 Fig — (A) Doubling time of the indicated strains during exponential growth in YESPL. Mean values and SDs from three independent experiments are shown. Not statistically significant (ns) differences were found (t-test, p>0.05). (B) Cell length of the indicated strains. Each point corresponds to the measurement of a single cell. Three independent experiments, each comprising of 100 cell measurements were performed. The red line indicates the mean value of the three independent biological experiments. Not statistically significant (ns) differences were found (Duncan’s new multiple range test, p>0.05). (C) Cell width measurements of the indicated strains were performed as described above for cell length. Not statistically significant (ns) differences were found (Duncan’s new multiple range test, p>0.05). (D) Optical microscopy images of FB2 and FB2Δhos2 cells during exponential growth in YEPSL liquid medium. (E) Quantification of the number of buds per cell in wild-type and Δhos2 mutants in the FB2 background. Mean values and SDs from three independent experiments are shown. Statistically significant differences are indicated (Fisher’s exact test, ** is used for p<0.01; **** for p<0.0001). (TIF) [file ppat.1005134.s004.tif]

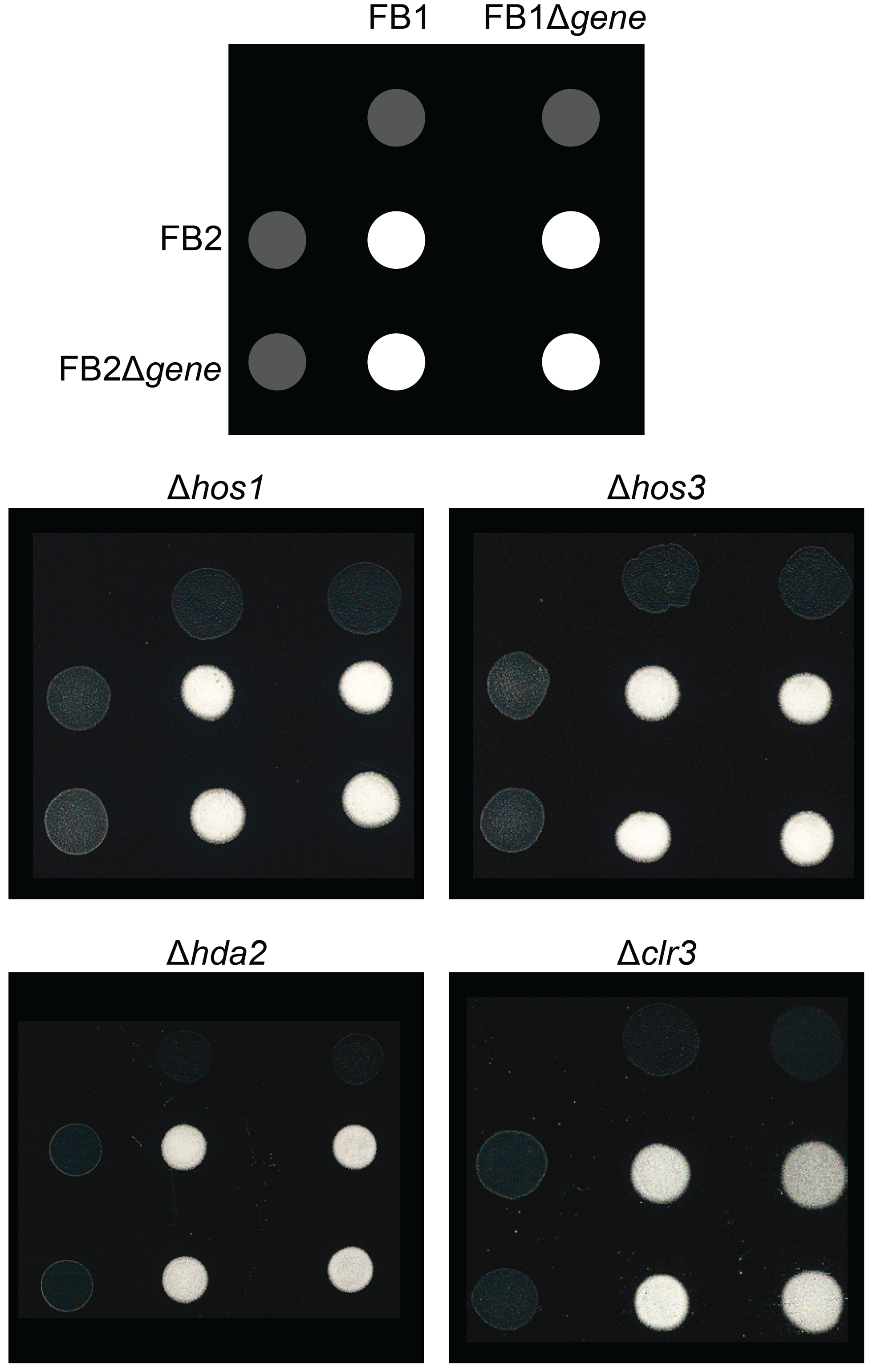

Supplement: S5 Fig — Indicated strains were grown in YEPSL medium to exponential phase and then spotted alone or in combination with other strains on PD charcoal plates and incubated for 24 hours at 25°C. (TIF) [file ppat.1005134.s005.tif]

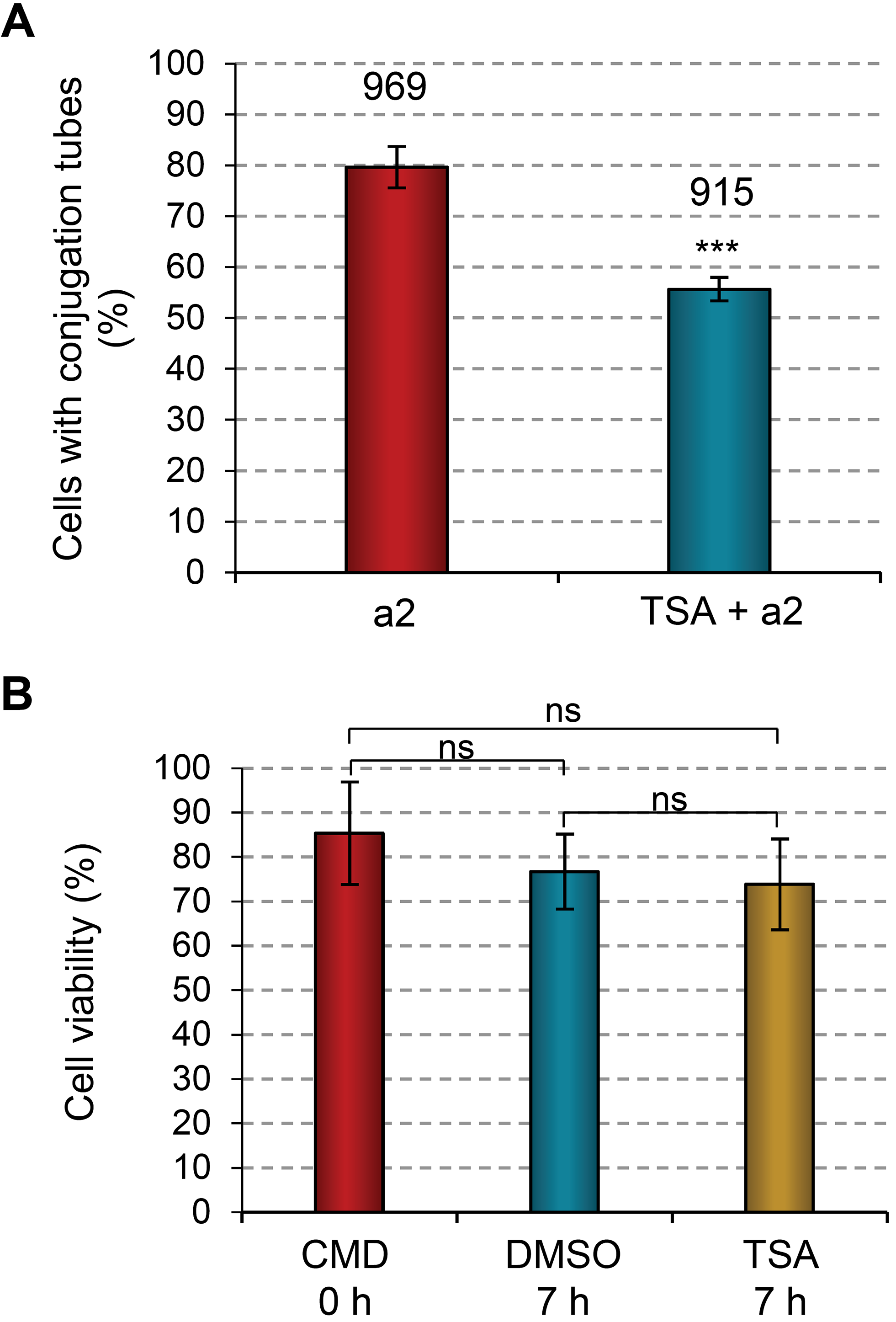

Supplement: S6 Fig — (A) Quantification of the effect of TSA on conjugation tube formation after a2 pheromone addition. FB1 cells were grown in CMD until exponential phase and treated either with 0.5 μg/ml of TSA for 2 hours, or with DMSO. a2 pheromone was then added to each culture for 5 hours before counting conjugation tube formation under the microscope. Mean values and SDs from three independent experiments are shown. Total number of cells counted is indicated above each column. *** denotes a statistically significant difference with p< 0.001 (t-test). (B) FB1 cells were grown in CMD until exponential phase and treated either with TSA to a final concentration of 0.5 μg/ml or DMSO for 7 hours. These cultures were assayed for cell viability, by plating 200 cells onto YPD plates and counting the number of colonies after 2 days incubation at 28°C. Mean values and SDs from three independent experiments are shown. Not statistically significant (ns) differences were found in any pairwise comparison (Duncan’s new multiple range test, p>0.05). (TIF) [file ppat.1005134.s006.tif]

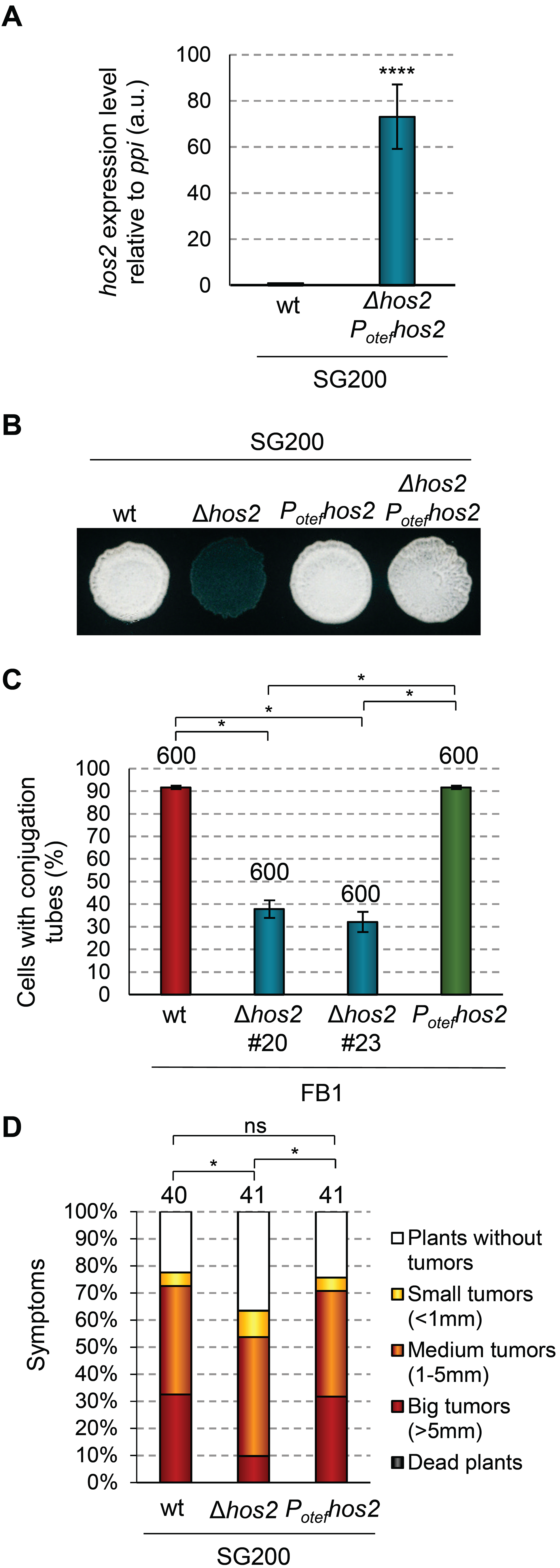

Supplement: S7 Fig — (A) Expression of hos2 relative to ppi in the indicated strains. Mean values and SDs from three independent experiments, each containing three technical replicates, are shown. Values are normalised to one of the biological replicates of the sample with the lowest expression value (SG200) that is assigned a value of 1. **** denotes a statistically significant difference with p<0.0001 (t-test) (B) Filamentation of the indicated solopathogenic strains grown on PD charcoal plates for 48 h at 25°C. (C) Quantification of the conjugation tube formation capacity of the indicated strains after 5 h exposed to a2 pheromone. Mean values and SDs from three independent experiments are shown. The total number of cells counted is indicated above each column. * denotes a statistically significant difference with p<0.05 (Duncan’s new multiple range test). (D) Quantification of plant symptoms infected with the indicated strains 14 days post-infection (dpi). Mean values of three independent experiments are shown. The total number of infected plants is indicated above each column. * denotes a statistically significant difference with p<0.05 (Mann-Whitney test) (TIF) [file ppat.1005134.s007.tif]

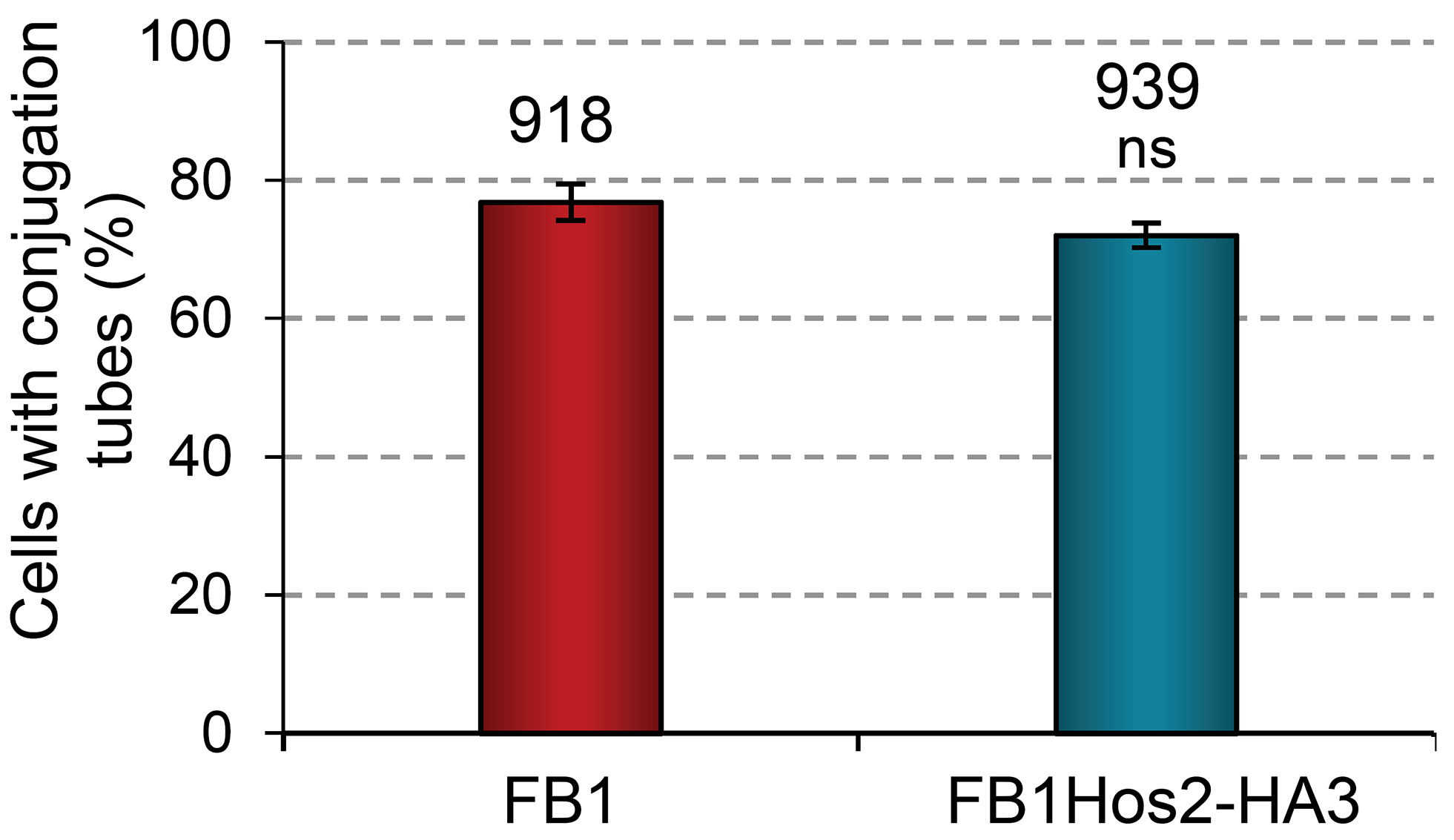

Supplement: S8 Fig — Exponentially growing cultures of the FB1 and FB1Hos2-HA3 strains were treated with a2 pheromone for 5 hours and conjugation tube formation quantified. Mean values and SDs from three independent experiments are shown. Total numbers of counted cells are indicated above each column. ns denotes not statistically significant difference (p>0.05, t-test). (TIF) [file ppat.1005134.s008.tif]

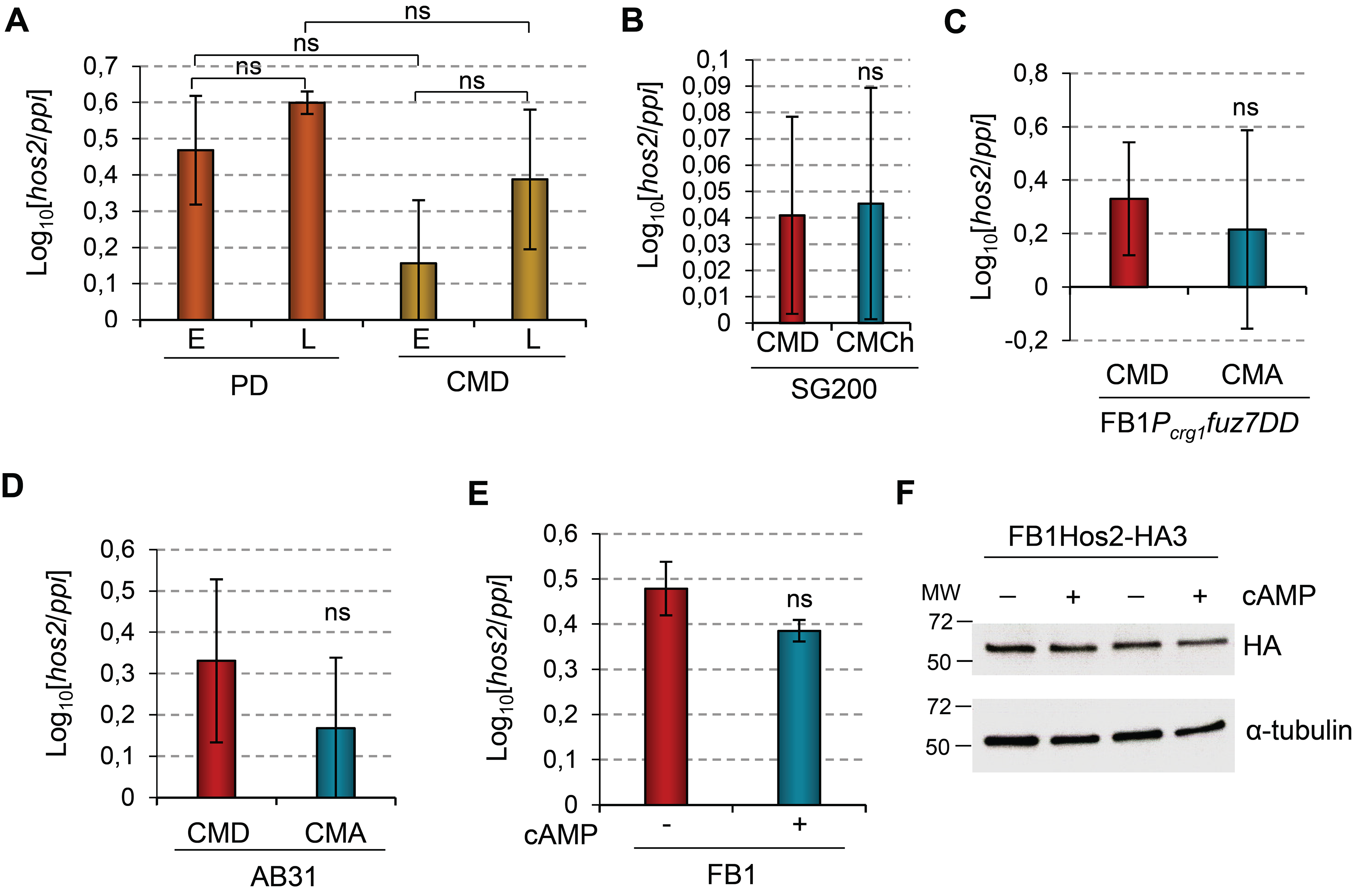

Supplement: S9 Fig — (A) hos2 expression level in the SG200 wild-type strain grown in CM or PD liquid media to early (E) or late (L) exponential phase. ns denotes not statistically significant difference (Duncan’s new multiple range test, p<0.05). (B) hos2 expression levels in the SG200 strain grown in CM liquid medium with 1% glucose as carbon source, or on CM charcoal plates. (C) hos2 expression upon activation of the MAPK pathway. Induction of the MAPKK Fuz7 was performed as described in the Methods section. (D) hos2 expression level upon induction of a compatible bE1/bW2 heterodimer in the AB31 background (see Methods). (E) Effect of cAMP addition on hos2 expression. 6 mM of cAMP was added to exponentially growing cultures of FB1 wild-type strain in PD broth. RNA extraction was performed 8 hours after cAMP addition. In A-E hos2 expression was quantified by RT-qPCR. Mean values and SDs from three independent experiments, each consisting of three technical replicates, are shown. In B-E ns denotes not statistically significant differences (t-test, p>0.05) (F) Hos2-HA3 protein levels from chromatin extracts used for ChIP, with or without the addition of cAMP. Tubulin was used as a loading control. (TIF) [file ppat.1005134.s009.tif]

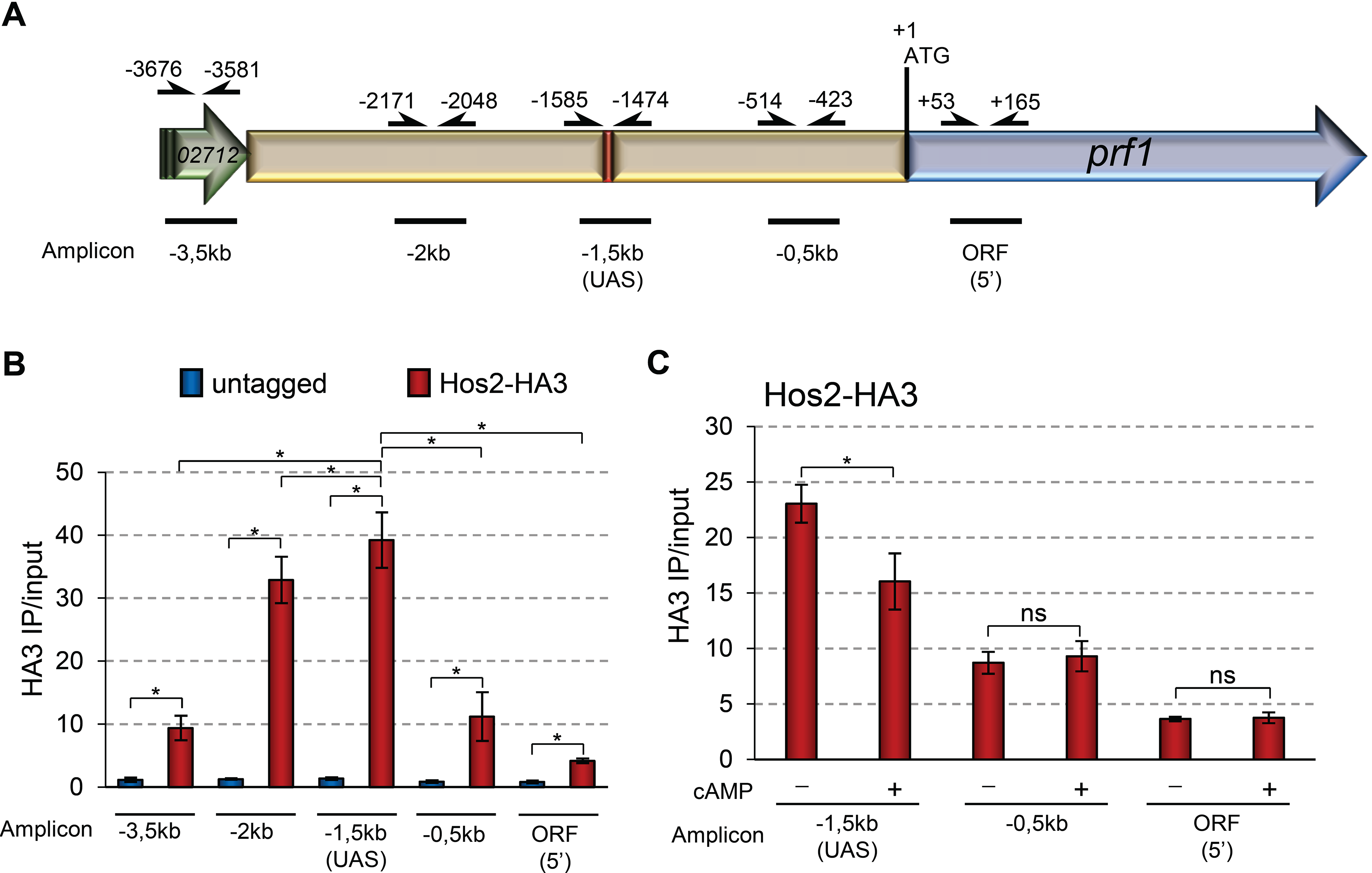

Supplement: S10 Fig — (A) Overview of the promoter and open reading frame of the prf1 gene. The probes used for qPCR analysis of the Hos2-HA3 ChIP experiment are indicated. Arrows represent the primers used for each specific amplicon. Numbers above the arrows indicate the specific coordinates relative to the first prf1 ORF ATG, with the adenine considered position +1. The name of each amplicon is indicated underneath and is used in subsequent panels of this figure. (B) ChIP analysis using an anti-HA antibody on chromatin extracts from either an untagged (blue) or a Hos2-HA3 (red) strain, grown in PD broth medium. Inmunoprecipitated DNA was analysed by qPCR, amplifying the regions indicated on the x axis and in panel A. Values correspond to the amount of DNA recovered by the HA IP divided by the amount of DNA in the corresponding input extract. Mean values and SDs from four independent experiments, each with three technical replicates, are shown. * denotes statistically significant differences (Duncan’s new multiple range test, p<0.05). (C) ChIP analysis was performed and analysed as in (A), except strains were grown in PD with or without the addition of 6 mM cAMP for 8 hours. For simplicity, values for the untagged strains are not shown, but were identical to those shown in (A) and did not vary upon cAMP addition. Statistically significant (*) and not significant (ns) differences are shown (Duncan’s new multiple range test, p<0.05). (TIF) [file ppat.1005134.s010.tif]
