# Supplementary material for: The Hos2 Histone Deacetylase Controls Ustilago maydis Virulence through Direct Regulation of Mating-Type Genes
Source: PLoS Pathog. 2015 Aug 28;11(8):e1005134. doi: 10.1371/journal.ppat.1005134 (PMC4552784; doi:10.1371/journal.ppat.1005134)
Supplement: S1 Table — (DOC) [file ppat.1005134.s011.doc]

S1 Table. *U. maydis* strains used in this study.

| **Strain** | **Relevant Genotype** | **Reference** |
| --- | --- | --- |
| FB1 | *a1 b1* | Banuett and Herskowitz (1989) |
| FB2 | *a2 b2* | Banuett and Herskowitz (1989) |
| SG200 | *a1 mfa2 bW2 bE1* | Bölker *et al* (1995) |
| HA103 | *a1* P*_hsp70_bW2* P*_otef_bE1* | Hartmann *et al* (1996) |
| AB31 | *a2 Pcrg:bW2 Pcrg:bE1* | Brachmann *et al* (2001) |
| FB1P*_crg1_fuz7^DD^* | *a1 P_crg1_:fuz7DD* | Müller *et al* (2003) |
| FB1Hos2-HA3 | *a1 b1 hos2-HA3:Hyg* | This work |
| FB1*P_otef_:hos2* | *a1 b1P_otef_:hos2:Cbx* | This work |
| FB1Δ*hos1* | *a1b1 Δhos1::Cbx* | This work |
| FB2Δ*hos1* | *a2b2 Δhos1::Cbx* | This work |
| FB1Δ*hos2* | *a1b1 Δhos2::Cbx* | This work |
| FB1Δ*hos2* | *a1b1 Δhos2::Nat* | This work |
| FB2Δ*hos2* | *a2b2 Δhos2::Cbx* | This work |
| FB1Δ*hos3* | *a1b1 Δhos3::Cbx* | This work |
| FB2Δ*hos3* | *a2b2 Δhos3::Cbx* | This work |
| FB1Δ*hda1* | *a1b1 Δhda1::Cbx* | This work, Reichmann *et al* (2001) |
| FB2Δ*hda1* | *a2b2 Δhda1::Cbx* | This work, Reichmann *et al* (2001) |
| FB1Δ*hda2* | *a1b1 Δhda2::Cbx* | This work |
| FB2Δ*hda2* | *a2b2 Δhda2::Cbx* | This work |
| FB1Δ*clr3* | *a1b1 Δclr3::Cbx* | This work |
| FB2Δ*clr3* | *a2b2 Δclr3::Cbx* | This work |
| SG200Δ*hda1* | *a1 mfa2 bW2 bE1* Δ*hda1::Hyg* | This work |
| SG200Δ*hda2* | *a1 mfa2 bW2 bE1* Δ*hda2::Hyg* | This work |
| SG200Δ*hos2* | *a1 mfa2 bW2 bE1* Δ*hos2::Hyg* | This work |
| SG200Δ*hos2* | *a1 mfa2 bW2 bE1* Δ*hos2::Nat* | This work |
| SG200*P_otef_:hos2* | *a1 mfa2 bW2 bE1 P_otef_:hos2:Cbx* | This work |
| SG200Δ*hos2P_otef_:hos2* | *a1 mfa2 bW2 bE1* Δ*hos2::Hyg P_otef_:hos2:Cbx* | This work |
| SG200Δ*clr3* | *a1 mfa2 bW2 bE1* Δ*clr3::Hyg* | This work |
| SG200Δ*tup1* | *a1 mfa2 bW2 bE1* Δ*tup1::Hyg* | Elías-Villalobos *et al* (2011) |
| SG200Δ*hda1Δhda2* | *a1 mfa2 bW2 bE1* Δ*hda1::Hyg Δhda2::Cbx* | This work |
| SG200Δ*hda1Δhos2* | *a1 mfa2 bW2 bE1* Δ*hda1::Hyg Δhos2::Cbx* | This work |
| SG200Δ*hda2Δhos2* | *a1 mfa2 bW2 bE1* Δ*hda2::Hyg Δhos2::Cbx* | This work |
| SG200Δ*hda1*Δ*hda2Δhos2* | *a1 mfa2 bW2 bE1* Δ*hda1::Hyg* Δ*hda2::Cbx* Δ*hos2::Nat* | This work |
| SG200Δ*hos2Δclr3* | *a1 mfa2 bW2 bE1* Δ*hos2::Hyg Δclr3::Cbx* | This work |
| SG200Δ*tup1*Δ*hos2* | *a1 mfa2 bW2 bE1* Δ*tup1::Hyg* Δ*hos2::Nat* | This work |
| FB1P*_crg1_fuz7^DD^*Δ*hos2* | *a1b1 P_crg1_:fuz7DD* Δ*hos2::Hyg* | This work |
| AB31Δ*hos2* | *a2b2 P_crg1_:bW2 P_crg1_:bE1* | This work |
