# Supplementary material for: The Hos2 Histone Deacetylase Controls Ustilago maydis Virulence through Direct Regulation of Mating-Type Genes
Source: PLoS Pathog. 2015 Aug 28;11(8):e1005134. doi: 10.1371/journal.ppat.1005134 (PMC4552784; doi:10.1371/journal.ppat.1005134)
Supplement: S2 Table — (DOC) [file ppat.1005134.s012.doc]

**S2 Table: Primers used in this study.**

| **Primer** | **Sequence (5’-3’)** | |
| --- | --- | --- |
| *hos1* deletion | | |
| UmHos1KO5-1 | | AACTTAATGGCACAACTTCG |
| UmHos1KO5-2 | | CACGGCCTGAGTGGCCCGAGACAATGTAGGCGACCC |
| UmHos1KO3-1 | | GTGGGCCATCTAGGCCGCGAGAGACTAGATTACATGC |
| UmHos1KO3-2 | | CAGATTGTCAAAATCCTACCC |
| *hos2* deletion | | |
| UmHos2KO5-1 | | GCAAGTGAGGTTGGCGATGC |
| UmHos2KO5-2 | | CACGGCCTGAGTGGCCACCATTATGGATGCGAACGG |
| UmHos2KO3-1 | | GTGGGCCATCTAGGCCAAGTCATTCGGGTTACCCTTGC |
| UmHos2KO3-2 | | TACAGATGCAAGCCATCTCC |
| *hos3* deletion | | |
| UmHos3KO5-1 | | TCGGCTGAGCATGAAAACTCC |
| UmHos3KO5-2 | | CACGGCCTGCGTGGCCGCTTCTGTACGAGACGGTGG |
| UmHos3KO3-1 | | GTGGGCCATCTAGGCCGCATCCTACGCAGTAACAGC |
| UmHos3KO3-2 | | GATTTTCAGAATGACCACAGG |
| *hda1* deletion | | |
| UmHda1KO5-1 | | AACTCGTAGATGACCGTCTAAGTGG |
| UmHda1KO5-2 | | CACGGCCTGAGTGGCCTCCGTTTGCACTTGTAGATGAACC |
| UmHda1KO3-1 | | GTGGGCCATCTAGGCCAACGCTTGATTGCAGTGACACAGTGC |
| UmHda1KO3-2 | | CTGCTTCTAAGAACGTCAGCAATTCC |
| *hda2* deletion | | |
| UmHda2KO5-1 | | AATGCGAGCGAAGCGAGATCAGACTTGC |
| UmHda2KO5-2 | | CACGGCCTGAGTGGCCAGCTGAGGACGACAAATGCGAGGATGG |
| UmHda2KO3-1 | | GTGGGCCATCTAGGCCAGGAGAGACGGTTGACACGAGAGTTTGG |
| UmHda2KO3-2 | | ACCAAGTGCAGTCGCTCTTTTATCAAGG |
| *clr3* deletion | | |
| UmClr3KO5-1 | | TTCAGAACACAGAACCGTCTCC |
| UmClr3KO5-2 | | CACGGCCTGAGTGGCCCGTTTCATTCGAGCGAATAGG |
| UmClr3KO3-1 | | GTGGGCCATCTAGGCCACATCCTCGCCCATAAGACCG |
| UmClr3KO3-2 | | ATGCTCGCAGGACAGTCAGG |
| *hos2* overexpression | | |
| UmHos2-ATGXmaISmaI | | aatcccgggCTGCAGGAATTCGATCCCATGGTTGTCATTGACTTTGACATGG |
| UmHos2-StopNotI | | aatgcggccgcCTATGACTTGACTTCTTCCTCGACCCAAGC |
| *hos2* qPCR | | |
| DHO946 | | TCCAATGCCACCTTTTCC |
| DHO947 | | AGGATGGTAGTAGCTGACG |
| HA3-tagging of Hos2 using the Gibson Assembly Kit | | |
| DHO915 | | AAGCTGGTACCGGGCCCCCCCTCGAGGCCAACGATTGCCATCAACTGGTCAGGCGGTCTGC |
| DHO916 | | ACGTCGTAGGGGTAGGCCGCGTTTGACTTGACTTCTTCCTCGACCCAAGC |
| DHO917 | | AAGCTGTGCGGCCGCATTAATAGGCCTGAGACCTCCCATGCTTTTCGACAAGCACG |
| DHO918 | | ACTATAGGGCGAATTGGAGCTCGGCCACTCAAGAGCTCGGTCAACGATATTCTCGGAC |
| DHO919 | | ATTGCCATCAACTGGTCAGGCGGTCTGC |
| DHO920 | | AAGAGCTCGGTCAACGATATTCTCGGAC |
| ChIP-qPCR | | |
| DHO981 | | TAGCAGATTGTTGGGAAACC |
| DHO982 | | TAGCCTTGACCTTGGCAAAGC |
| DHO983 | | ATGCAACCGACTTATTGTCC |
| DHO984 | | TGAACCGCATTAGATTTCCAGG |
| DHO985 | | ATCAGATCATGGTCGACCTC |
| DHO986 | | AGAGGCTGATCGCTTGATTGG |
| DHO991 | | TCTCGCTCATAAGCTTCTTGG |
| DHO992 | | ATCTTTTGTTGCAGCTCTCG |
| DHO1001 | | TCTTACGCTCTCTTGATGG |
| DHO1002 | | ACTTCGTCGATCTTGAAAGC |
| DHO1024 | | TAACTTCCAGCAGCTCCACG |
| DHO1025 | | TTACGGCAGTTGTACGTTCC |
